# Chronic stress induces depression through MDGA1-Neuroligin2 mediated suppression of inhibitory synapses in the lateral habenula

**DOI:** 10.7150/thno.104282

**Published:** 2025-01-02

**Authors:** Xuehui Wang, Hao Wei, Zhe Hu, Jie Jiang, Xinyan Dong, Jinpiao Zhu, Haiyan Chen, Nils Brose, Noa Lipstein, Tonghui Xu, Steven A. Connor, Daqing Ma, Yicheng Xie

**Affiliations:** 1 Perioperative and Systems Medicine Laboratory, Department of Anesthesiology, Children's Hospital, Zhejiang University School of Medicine, National Clinical Research Center for Child Health, Hangzhou, 310052, Zhejiang Province, China.; 2 Department of Biology, York University, 4700 Keele Street, Toronto, ON, M3J 1P3, Canada.; 3 Department of Molecular Neurobiology, Max Planck Institute for Multidisciplinary Sciences, Göttingen, 37075, Germany.; 4 Department of Molecular Physiology and Cell Biology, Leibniz-Forschungsinstitut für Molekulare Pharmakologie and NeuroCure Excellence Cluster, Berlin, 13125, Germany.; 5 Department of Laboratory Animal Science, Fudan University, Shanghai, 200032, China.; 6 Division of Anesthetics, Pain Medicine & Intensive Care, Department of Surgery and Cancer, Faculty of Medicine, Imperial College London, Chelsea and Westminster Hospital, London SW10 9NH, United Kingdom.

**Keywords:** Nlgn2, MDGA1, Inhibitory synapses, Lateral habenula, Stress, Depression

## Abstract

**Rationale:** The hyperactivity of lateral habenula (LHb) has been implicated in the pathophysiology of depression, but the regulatory mechanisms of inhibitory synapses in this context remains unclear. MDGA1 and neuroligin2 (Nlgn2), both regulators of inhibitory synapses, selectively interact in the LHb. We aimed to investigate if their interaction contributes to chronic restrained stress (CRS)-induced depression by modulating inhibitory synapses.

**Methods:** Transgenic mouse models were established to conditional knockout/recover of MDGA1 expression or knockin Nlgn2 variant incapable of binding MDGA1 in the LHb, using viral Cre-recombinase expression. Synaptic function and density were assessed through electrophysiology and immunostaining, respectively. An acute restrained stress (ARS) model and chemogenetic activation of the lateral hypothalamus (LH) were used to stimulate the LHb. Behavioral tests related to depression were conducted following CRS.

**Results:** MDGA1 and Nlgn2 selectively interacted in the LHb, which was elevated following CRS. Germline knockout of MDGA1 increased inhibitory transmission and GABAergic synapse density in the LHb, effects that were reversed by adult re-expression of MDGA1. Introduction of the Nlgn2 variant incapable of binding MDGA1 similarly enhanced inhibitory transmission and increased GABAergic synapse density in the LHb. Both germline MDGA1 deficiency and introduction of the Nlgn2 variant mitigated ARS- and LH activation-induced LHb neuron hyperactivation. MDGA1 deficiency in the LHb during adulthood increased inhibitory synaptic strength and conferred significant resistance to CRS-induced depressive behaviors, similar to the effects of introducing the Nlgn2 variant in the LHb.

**Conclusions:** Our findings suggests that MDGA1-mediated suppression of Nlgn2 facilitates depression onset through limiting GABAergic synapse formation within the LHb. Targeting MDGA1/Nlgn2 complexes residing at GABAergic synapses within the lateral habenula may be viable for alleviating core behavioral symptoms of major depression.

## Introduction

Chronic stress is well documented as a risk factor for the development of major depression 1-3. Similar to humans, rodents exposed to chronic stress demonstrate behavioral changes consistent with depression, including anhedonia, social withdrawal, cognitive impairments and demotivation 4-6. The onset of major depression following stress has been linked to maladaptive changes in specific brain regions and/or circuits. Among these, the lateral habenula (LHb) has emerged as a key brain region in the pathophysiology of stress-induced depression 7, 8. Evidence from both human 9-11 and animal studies demonstrated hyperactivity and synaptic remodeling in LHb associated with the onset of major depression-like symptoms following stress 7, 8, 12-15. Additionally, aberrant LHb activity is associated with selective features of major depression such as learned helplessness 12, 16 and anhedonia 17, which are observed in parallel with upregulated neuronal activity specifically within the LHb.

Although considerable evidence implicates the LHb in the pathogenesis of major depression, the underlying molecular mechanisms that drive hyperexcitation within this brain region remain ill defined. Exploring these mechanisms would therefore advance our understanding of the molecular and cellular basis for major depression and help identify clinically relevant treatment targets. Synapse organizing proteins serve as molecular codes that determine the development, composition and function of synapses 18-23. The importance of these molecular constraints is demonstrated by a growing body of genetic studies linking synapse organizers to neurodevelopmental and neuropsychiatric disorders, including major depression 24-27.

Many synapse organizers, including the well-characterized neuroligins (Nlgns), are synaptogenic in nature 22, 28-30. Localized primarily to postsynaptic sites, Nlgns bind to presynaptic neurexins (Nrxns) to drive the recruitment of cellular machinery required for synapse maturation and validation 31-33. Humans and rodents have 5 and 4 Nlgn isoforms respectively, which form homomeric and heteromeric dimers that determine synapse identity and properties in a variant-dependent fashion 30, 34. Whereas Nlgn1 promotes glutamatergic synapses 29, Nlgn2 preferentially localizes to inhibitory synapses 35, 36, where it enhances the development and refinement of GABAergic synapses. Moreover, Nlgn2 plays an ongoing role in maintaining the function of GABAergic synapses during adulthood, as virally mediated deletion of *Nlgn2* in the medial prefrontal cortex (mPFC) or lateral septum (LS) in adult mice led to reduced inhibitory synaptic transmission 37, 38, resembling the deficit phenotype in constitutive *Nlgn2* knockout mice. Nlgn2 interacts on the postsynaptic side with inhibitory scaffolds, including gephyrin, collybistin and Slitrk3 35, 39.

Nlgn-Nrxn complex formation is regulated by a family of synapse suppressors known as MAM domain-containing GPI anchors (MDGAs) 19, 40-46. *In vivo*, MDGA1 primarily suppresses inhibitory synapses whereas MDGA2 represses excitatory synapses, providing further regulatory oversite of synapse development and properties. MDGAs limit Nlgn function through direct steric hindrance, binding and occluding Nrxn binding sites on Nlgns 47-49. MDGA1 is expressed in hippocampal pyramidal neurons where it selectively limits perisomatic inhibitory synapses formation in CA1 pyramidal cells 40, suggesting a role in repressing Nlgn2. Moreover, pull-down assays utilizing detergent-solubilized mouse brain membrane fractions demonstrated enrichment of MDGA1 by Nlgn2, but not by Nlgn1. This suggests that while the overall mechanisms of MDGAs binding to Nlgns are identical, there may be unidentified processes that mediate the selective association of MDGA1 with Nlgn2, rather than Nlgn1, *in vivo* 49.

Here, we sought to determine if synaptic remodeling associated with stress-induced depression in the LHb alters MDGA1-Nlgn2 complex formation and associated synaptic function. We further probed if altering MDGA1-Nlgn2 interactions affects measures of depression-like behaviors in mouse models, and whether preventing this interaction reverses these effects. To explore these questions, we combined genetic manipulations of MDGA1/Nlgn2 with functional assessment of LHb synapses in mice subjected to restraint stress and subsequent biobehavioral assays for depression.

## Results

### MDGA1 and Nlgn2 are expressed and co-localize within the LHb, which increases following chronic stress

**Figure 1:**
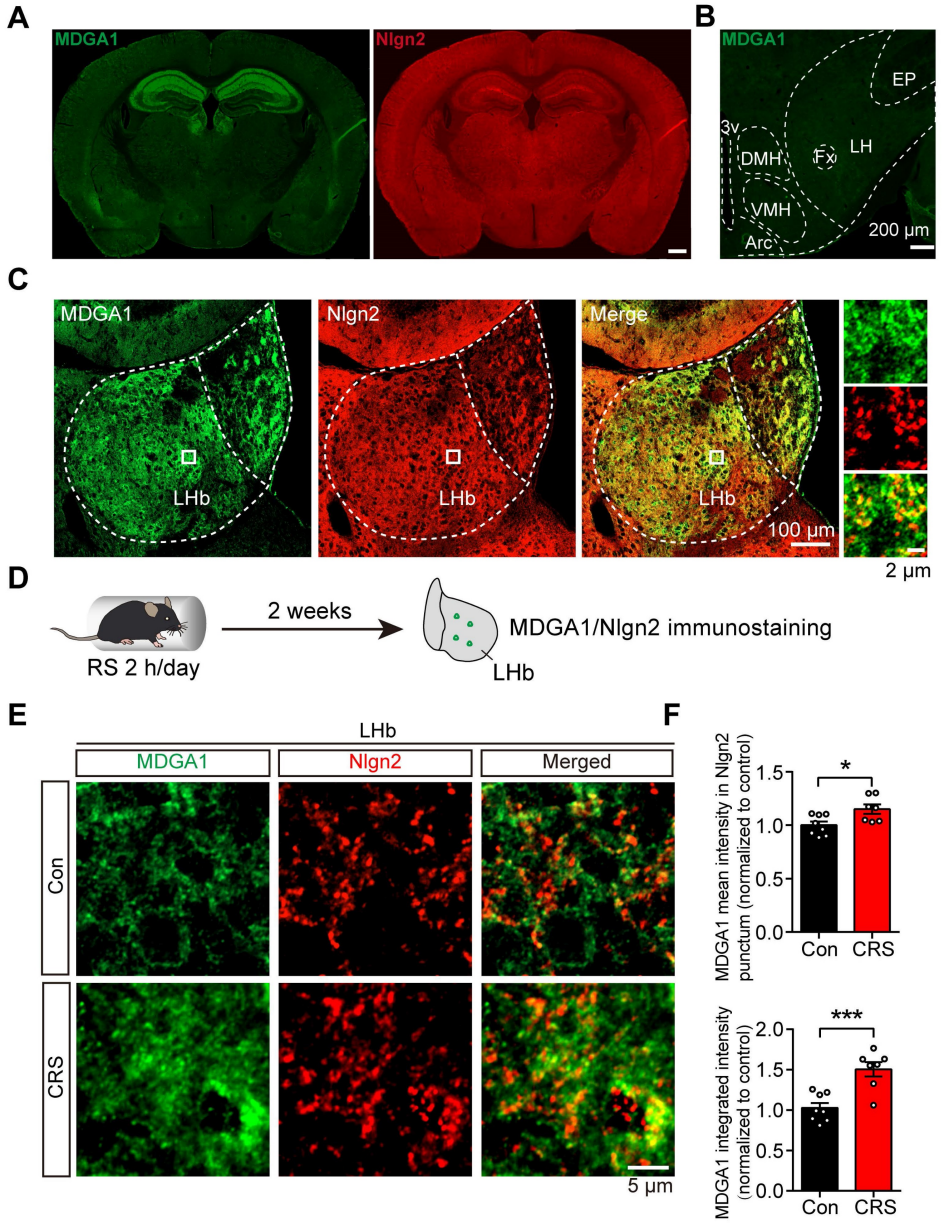
** MDGA1 and Nlgn2 co-localize within the LHb, which is increased following CRS. (A)** Representative MDGA1 and Nlgn2 immunofluorescence staining images of mouse coronal brain sections. **(B)** MDGA1 immunofluorescence staining in the lateral hypothalamus.** (C)** Representative MDGA1 and Nlgn2 immunofluorescence staining images in the LHb. **(D)** Schematic diagram of the experimental procedure.** (E)** High magnification photomicrographs obtained from the LHb of control and CRS mice showing MDGA1 (green) and Nlgn2 (red) labeling. **(F)** The amount of MDGA1 colocalized with Nlgn2 in the LHb is significantly increased following CRS (top graph) and the expression of MDGA1 in LHb is also increased following CRS (bottom graph). n = 8 mice for control group, n = 7 mice for CRS group; unpaired t test, *p* = 0.0166 (top), *p* = 0.0006 (bottom). Data are presented as the mean ± SEM. *p < 0.05, ***p < 0.001.

Previous studies showed that MDGA1 selectively interacts with Nlgn2 to regulate the development of inhibitory synapses 40. However, whether this interaction takes place in the LHb to regulate inhibitory synapses remains unclear. We first sought to establish the expression profile of MDGA1 and Nlgn2 within the LHb using immunofluorescent staining. MDGA1 was highly expressed in both the hippocampus and lateral habenula (LHb), but was almost absent in the lateral hypothalamus (LH) (Figure 1A, B), which provides physiologically relevant inputs onto the LHb that are engaged in response to stress 15. In contrast, Nlgn2 was also present in the LHb, but was relatively homogeneously distributed throughout the brain (Figure 1B). Given that both MDGA1 and Nlgn2 were present in the LHb, we next assessed the co-localization of MDGA1 and Nlgn2 in this region. Immunolabeling results revealed colocalization of MDGA1 and Nlgn2 in the LHb (Figure 1C). Given the essential role of the LHb in regulation of depression, we next assessed whether the expression patterns of MDGA1 and Nlgn2 were changed using a mouse model of chronic stress, which is a major risk factor for depression onset. After subjecting mice for 14 consecutive days of 2 h per day chronic restraint stress (CRS) (Figure 1D), the amount of MDGA1 detected including the amount specifically colocalized with Nlgn2 in the LHb was significantly increased (Figure 1E, F). Given that Nlgn2 plays an ongoing role in maintaining the function of GABAergic synapses, we investigated the effects of CRS and acute restraint stress (ARS) on inhibitory synaptic transmission in LHb neurons by performing whole-cell recordings in brain slices. The results showed that ARS exerted little effect on mIPSC and CRS significantly decreased the mIPSC frequency but not amplitude (Supplementary Figure S1A-C), which were consistent with the results of a previous study 15. Taken together, these results indicates that the interaction between MDGA1 and Nlgn2 in the LHb may play roles in the regulation of LHb function in the development of stress-induced depression.

### MDGA1 deficiency elevates inhibitory synaptic strength in pyramidal neurons in the LHb

**Figure 2:**
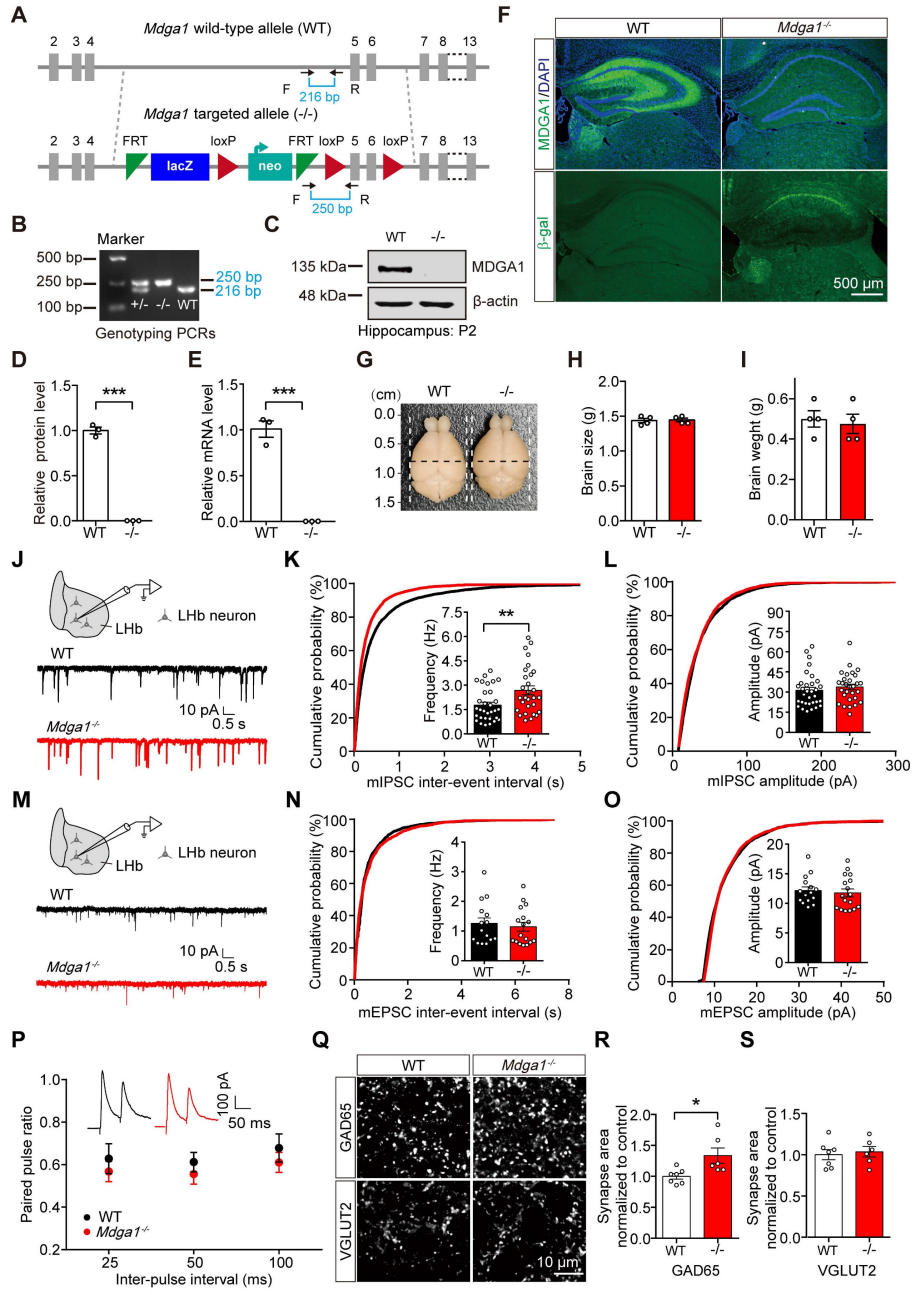
** MDGA1 deficiency increases inhibitory but not excitatory synaptic inputs onto neurons in the LHb. (A)** Strategy for generation of *Mdga1*^-/-^ mice, β-galactosidase (LacZ) and neomycin (neo) gene fragments are inserted between exons 4 and 5 of MDGA1. Black arrows indicate forward and reverse primers used for genotyping. **(B)** PCR genotyping of genomic DNA from *Mdga1^+/-^*, *Mdga1*^-/-^ and WT mice using forward and reverse primers which flank the loxp site and show a wild-type (WT) band at 216 bp and a flox-inserted band at 250 bp. **(C)** Representative immunoblots of hippocampal lysates. **(D)** Quantification of protein levels from **(C)**. **(E)** Quantitative RT-PCR analyses of MDGA1 mRNA levels in WT and *Mdga1*^-/-^ mice.** (F)** MDGA1 and β-galactosidase staining of WT and *Mdga1*^-/-^ mouse brain. The inserted LacZ is expressed from the *Mdga1* locus.** (G-I)** Representative images of whole brains and quantification of brain size and weights of WT and *Mdga1*^-/-^ mice at P56. **(J)** Schematic showing patch-clamp recording performed in the neurons of LHb (top) and representative mIPSC traces (bottom).** (K)** *Mdga1*^-/-^ neurons exhibited a significant increase in mIPSC frequency relative to WT. n = 32 neurons, from 4 mice for WT; n = 29 neurons, from 4 mice for *Mdga1*^-/-^; unpaired t test, *p* = 0.0074.** (L)** mIPSC amplitude did not differ significantly between groups. Unpaired t test, *p* = 0.4255.** (M-O)** Schematic showing patch-clamp recordings performed in the neurons of LHb (top) and representative mEPSC traces (bottom)** (M)**. Neither the frequency** (I**; unpaired t test, *p* = 0.6427**)** nor the amplitude **(O**; unpaired t test, *p* = 0.6613**)** was altered in *Mdga1*^-/-^ neurons relative to WT. n = 15 neurons, from 4 mice for WT; n = 17 neurons, from 4 mice for *Mdga1*^-/-^.** (P)** Paired pulse ratios plotted against inter-stimulus intervals. Representative traces of eIPSC paired-pulse stimulation at a 50 ms interval are shown (inset). There was not a significant difference between groups. n = 17 neurons, 4 mice for both genotypes; two-way ANOVA, p = 0.1743, F _(1, 96)_ = 1.8730.** (Q)** Representative confocal images from LHb immunolabeled for GAD65 and VGLUT2. **(R)** Quantification of punctate integrated intensity per tissue area showed a significant increase in GAD65 in the LHb of *Mdga1*^-/-^ mice. n = 7 mice for WT group, n = 6 mice for *Mdga1*^-/-^ group; unpaired t test, *p* = 0.0199.** (S)** Quantification of punctate integrated intensity per tissue area showed no changes in VGLUT2 in the LHb of *Mdga1*^-/-^ mice. n = 7 mice for WT group, n = 6 mice for *Mdga1*^-/-^ group; unpaired t test, *p* = 0.6985. Data are presented as the mean ± SEM. *p < 0.05, **p < 0.01, unpaired t test.

To evaluate the function of MDGA1 in the brain, the endogenous gene was disrupted in transgenic knock-in mice, in which β-galactosidase (LacZ) and neomycin (neo) gene fragments were inserted between exons 4 and 5 (Figure 2A, B). Immunoblots demonstrated that the expression of MDGA1 was abolished in *Mdga1*^-/-^ mice (Figure 2C, D). Quantitative RT-PCR analyses also showed that *Mdga1* mRNA was completely eliminated in *Mdga1*^-/-^ mice (Figure 2E). Moreover, immunofluorescent staining showed that MDGA1 signal was not detectable in the hippocampus or habenula of *Mdga1*^-/-^ mice compared with WT mice (Figure 2F). Expression of β-galactosidase (β-gal) from the *Mdga1* locus showed that β-gal was expressed in the hippocampus and habenula of *Mdga1*^-/-^ mice, while no fluorescence signal was detected in that of WT mice (Figure 2F). In addition, *Mdga1*^-/-^ mice showed no difference in brain size and weight when compared with littermate WT mice (Figure 2G-I).

Previous studies indicated that MDGA1 is primarily expressed in excitatory neurons in the hippocampus 40, but the cell specific expression of MDGA1 in other brain regions remains unclear. We performed immunofluorescence of β-gal and a neuronal marker (NeuN) in the β-gal knock-in mice and found almost all β-gal positive cells co-localized with NeuN (Supplementary Figure S1A, B). Co-staining for the excitatory neuron marker CaMKII or interneuron subtype marker parvalbumin (PV) revealed that most β-gal-labeled cells were CaMKII positive (92.87%), while no overlap was observed between PV and β-gal in the LHb (Figure S2A, C). These results indicate that MDGA1 is primarily expressed in excitatory neurons within the LHb.

To determine whether MDGA1 deficiency altered basal GABAergic transmission, we measured mIPSCs in LHb neurons. Compared with control littermates, mIPSC frequency, but not amplitude, was increased in the LHb of the *Mdga1^-/-^* mice (Figure 2J-L). Conversely, no changes in mEPSC frequency or amplitude were detected in LHb neurons from *Mdga1^-/-^* mice (Figure 2M-O). The increased frequency of mIPSCs suggests that MDGA1 deficiency may selectively enhance the function or density of GABAergic pre-synaptic terminals in the LHb. However, no differences were found in paired pulse ratio (PPR) of evoked IPSCs (Figure 2P), indicating the altered mIPSC frequency may be attributed to enhanced synapse numbers. Therefore, we next assessed synapse numbers by measuring the inhibitory presynapse marker GAD65, as well as the excitatory presynapse marker vesicular glutamate transporter 2 (VGLUT2). The punctate immunofluorescence of GAD65 was significantly increased, while quantitation of VGLUT2 revealed no changes in the LHb of *Mdga1*^-/-^ mice (Figure 2Q-S). These results suggest that enhanced basal inhibitory transmission may reflect an increase in GABAergic synapse density in the LHb in *Mdga1^-/-^* mice.

### Conditional KO of *Mdga1* in the LHb increases inhibitory synaptic transmission and GABAergic synapse density

**Figure 3:**
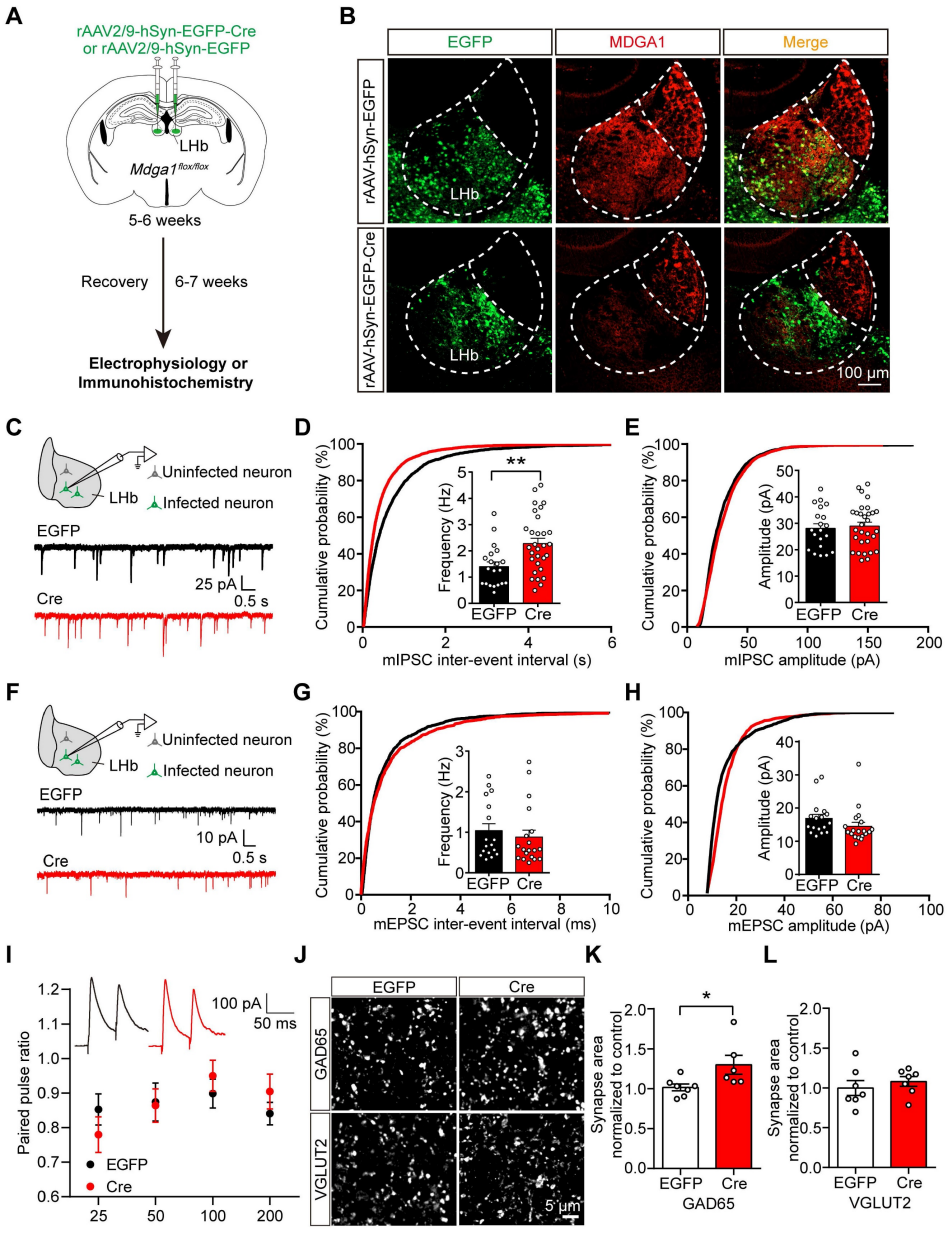
** Conditional KO of *Mdga1* in the LHb increases inhibitory synaptic transmission and GABAergic synaptic density. (A)** Schematic diagram of the experimental procedure. **(B)** Immunohistochemical staining of MDGA1 in each group shows that MDGA1 expression was significantly decreased in the LHb after Cre-recombinase expression. **(C)** Schematic diagram of recording (top) and representative mIPSCs traces (bottom). **(D)** KO neurons exhibited a significant increase in mIPSC frequency relative to control EGFP neurons. n = 20 neurons, from 4 mice for EGFP, n = 30 neurons, from 6 mice for Cre; unpaired t test, *p* = 0.0034.** (E)** mIPSC amplitude did not differ between the Cre and EGFP groups. n = 20 neurons, from 4 mice for EGFP, n = 30 neurons, from 6 mice for Cre; unpaired t test, *p* = 0.1332.** (F)** Schematic diagram of recording procedure (top) and representative mEPSCs traces (bottom) **(G)** mEPSC frequency did not differ between the Cre and EGFP groups. n = 17 neurons, from 3 mice for EGFP, n = 19 neurons, from 4 mice for Cre; unpaired t test, *p* = 0.5251.** (H)** mEPSC amplitude did not differ between the Cre and EGFP groups. n = 17 neurons, from 3 mice for EGFP, n = 19 neurons, from 4 mice for Cre; unpaired t test, *p* = 0.1799.** (I)** Paired-pulse ratio of evoked IPSCs was not significantly different between groups. eIPSC paired-pulse ratio (PPR) traces at a 50 ms interval (inset). n = 12 neurons, from 3 mice for each group; two-way ANOVA,* p* = 0.8085, F _(1, 88)_ = 0.0591.** (J)** Representative confocal images from LHb of EGFP and Cre brain sections immunolabeled with inhibitory presynaptic marker GAD65 and excitatory presynaptic marker VGLUT2 are shown. **(K)** Quantification of punctate integrated intensity per tissue area shows a significant increase in GAD65 in LHb of Cre mice. n = 7 mice for EGFP group, n = 6 mice for Cre group; unpaired t test, *p* = 0.0354.** (L)** Quantification of punctate integrated intensity per tissue area showed no change in VGLUT2 in LHb of Cre mice. n = 7 mice for EGFP group, n = 6 mice for Cre group; unpaired t test, *p* = 0.4821. Data are presented as the mean ± SEM. *p < 0.05, **p < 0.01, unpaired t test.

To further investigate the role of MDGA1 in the LHb, we stereotaxically delivered rAAV2/9-hSyn-EGFP-Cre or rAAV2/9-hSyn-EGFP (as a control) bilaterally to the LHb of adult *Mdga1*^*flox/flox*^ mice to delete *Mdga1* selectively in the LHb neurons (Figure 3A). MDGA1 signal remained intact in the LHb of *Mdga1*^flox/flox^ mice infected with rAAV2/9-hSyn-EGFP, but was substantially reduced in the LHb of *Mdga1*^flox/flox^ mice infected with rAAV2/9-hSyn-EGFP-Cre, confirming deletion of *Mdga1* in LHb neurons (Figure 3B).

To determine the contribution of MDGA1 to maintain synaptic function in the LHb during adulthood, we performed whole-cell voltage clamp recordings from virus-infected LHb neurons in acute brain slices after 6-7 weeks of viral expression (Figure 3C). We found mIPSC frequency was significantly increased in the virus-infected neurons in *Mdga1*-cKO mice relative to controls (Figure 3D), with no significant changes in mIPSC amplitude between groups (Figure 3E). Consistent with these effects, a significant leftward shift in the cumulative probability curve of the mIPSC frequency was observed (Figure 3D, E). mEPSC amplitude and frequency were comparable between the *Mdga1*-cKO and control mice (Figure 3F-H). The PPR of evoked IPSCs remained unchanged between groups (Figure 3I). Thus, the reduction of MDGA1 in LHb neurons selectively enhanced inhibitory but not excitatory synaptic transmission.

To investigate the contribution of MDGA1 in maintaining synaptic density in the LHb during adulthood, we further assessed the quantitation of GAD65 and VGLUT2 in the LHb by immunolabeling, representing GABAergic and glutamatergic presynaptic terminals respectively. The quantitation revealed that punctate immunofluorescence of GAD65 was significantly increased in the LHb in the *Mdga1*-cKO mice relative to WT mice (Figure 3J, K), with no change in VGLUT2 punctate immunofluorescence (Figure 3J, L). Thus, deletion of *Mdga1* in LHb neurons during adulthood also increased inhibitory but not excitatory synapse numbers, suggesting that MDGA1 regulates GABAergic synapses in mature LHb neurons.

### Restoring MDGA1 expression in the LHb decreases inhibitory synaptic transmission

**Figure 4:**
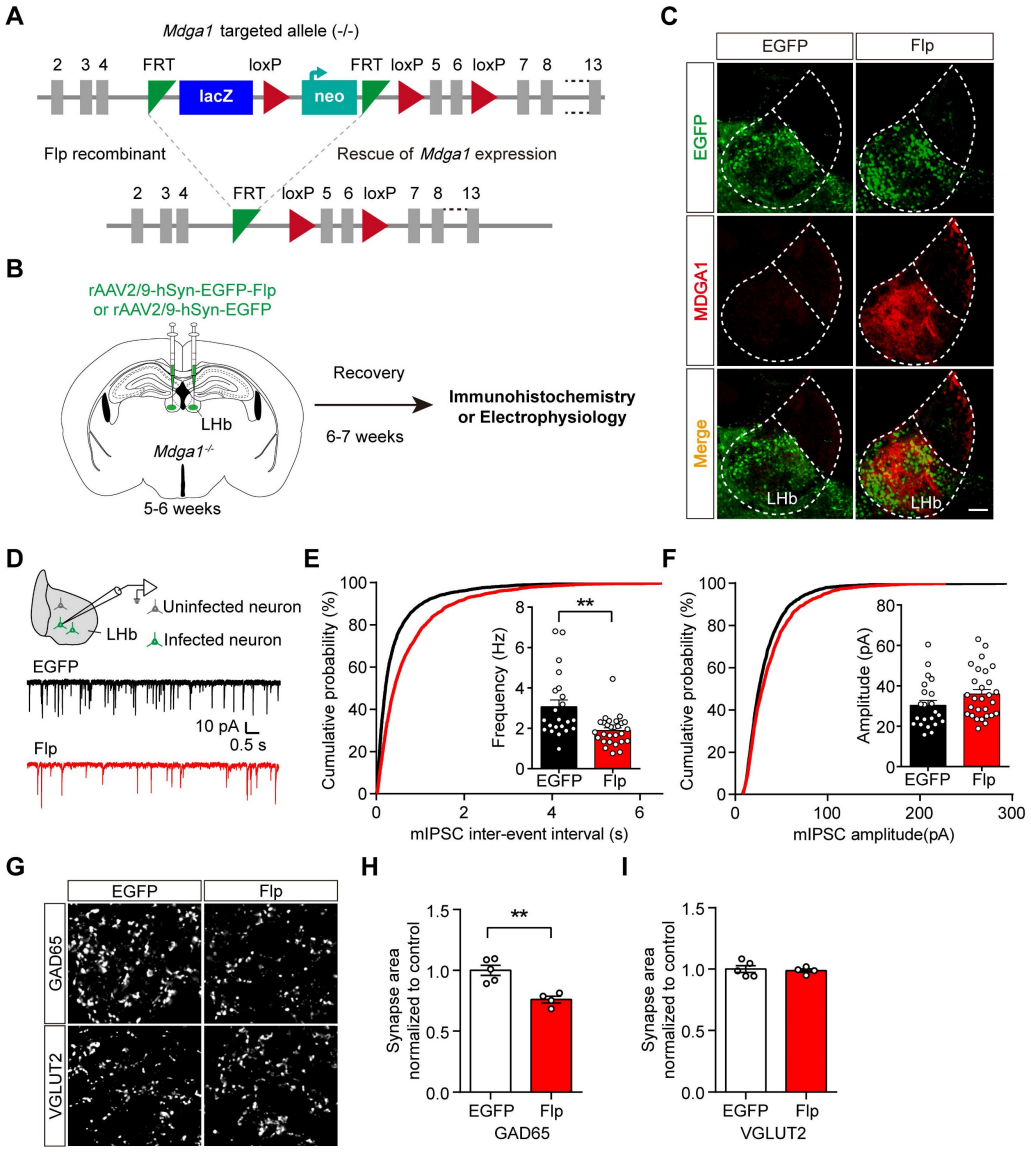
** Rescuing MDGA1 expression in adult *Mdga1* KO LHb decreases inhibitory synaptic strength. (A)** Strategy used to re-express MDGA1 in *Mdga1^-/-^* mice. **(B)** Schematic diagram of the experimental procedure is shown. **(C)** Immunohistochemical staining of MDGA1 showed that MDGA1 was re-expressed in *Mdga1^-/-^* mice infected with rAAV-hSyn-EGFP-Flp in LHb compared to *Mdga1^-/-^* mice infected with rAAV-hSyn-EGFP. Scale bar, 100 μm. **(D)** Schematic diagram of recording procedure (top) and representative mEPSCs traces (bottom). **(E)** Rescuing MDGA1 in LHb decreased mIPSC frequency. n = 22 neurons, from 4 mice for EGFP, n = 28 neurons, from 4 mice for Flp; unpaired t test, *p* = 0.0011. **(F)** Rescuing MDGA1 in LHb did not alter mIPSC amplitude. n = 22 neurons, from 4 mice for EGFP, n = 28 neurons, from 4 mice for Flp; unpaired t test, *p* = 0.1095.** (G)** Representative confocal images from LHb immunolabeled for GAD65 and VGLUT2. **(H)** Quantification of punctate integrated intensity per tissue area showed a significant decrease in GAD65 in the LHb of Flp mice. n = 5 mice for EGFP group, n = 4 mice for Flp group; unpaired t test, *p* = 0.0028.** (I)** Quantification of punctate integrated intensity per tissue area shows no changes in VGLUT2 in the LHb of Flp mice. n = 5 mice for EGFP group, n = 4 mice for Flp group; unpaired t test, *p* = 0.7250. Data are presented as the mean ± SEM. **p < 0.01, unpaired t test.

To test the sufficiency of MDGA1 in regulating inhibitory synaptic transmission in the adult LHb, we reintroduced MDGA1 into LHb neurons of *Mdga1^-/-^* mice during adulthood through rAAV expression of Flp to remove the disruptive LacZ insertion (Figure 4A, B). rAAV2/9-hSyn-EGFP-Flp or rAAV2/9-hSyn-EGFP (as a control) were injected into the LHb of *Mdga1^-/-^* mice at the age of 5-6 weeks. As shown in Figure 4C, after 6-7 weeks of viral expression, the MDGA1 signal was rescued in the LHb of *Mdga1^-/-^* mice infected with rAAV2/9-hSyn-EGFP-Flp, but was undetectable in the LHb of *Mdga1^-/-^* mice infected with rAAV2/9-hSyn-EGFP (Figure 4C), confirming MDGA1 re-expression in the LHb of *Mdga1^-/-^* mice.

We next investigated whether reintroducing MDGA1 in the LHb of *Mdga1^-/-^* mice reduced inhibitory synaptic transmission. As shown in Figure 4D-F, mIPSC frequency was significantly decreased in the rAAV2/9-hSyn-EGFP-Flp infected neurons in the LHb of *Mdga1^-/-^* mice relative to that in rAAV2/9-hSyn-EGFP infected LHb neurons of *Mdga1^-/-^* mice, whereas mIPSC amplitude was not significantly different between groups. To further test if inhibitory synapse numbers were changed following reconstitution of MDGA1, LHb neurons were immunolabeled for GAD65 and VGLUT2. Quantification of punctate intensity revealed a significant decrease in GAD65 in the LHb of rAAV2/9-hSyn-EGFP-Flp infected mice, whereas, VGLUT2 remained unchanged (Figure 4G-I). Thus, restoring MDGA1 expression in the LHb during adulthood reduced inhibitory synaptic transmission and inhibitory synapse density.

### *Mdga1* KO attenuates ARS- or LH activation-induced LHb neuron hyperactivation

**Figure 5:**
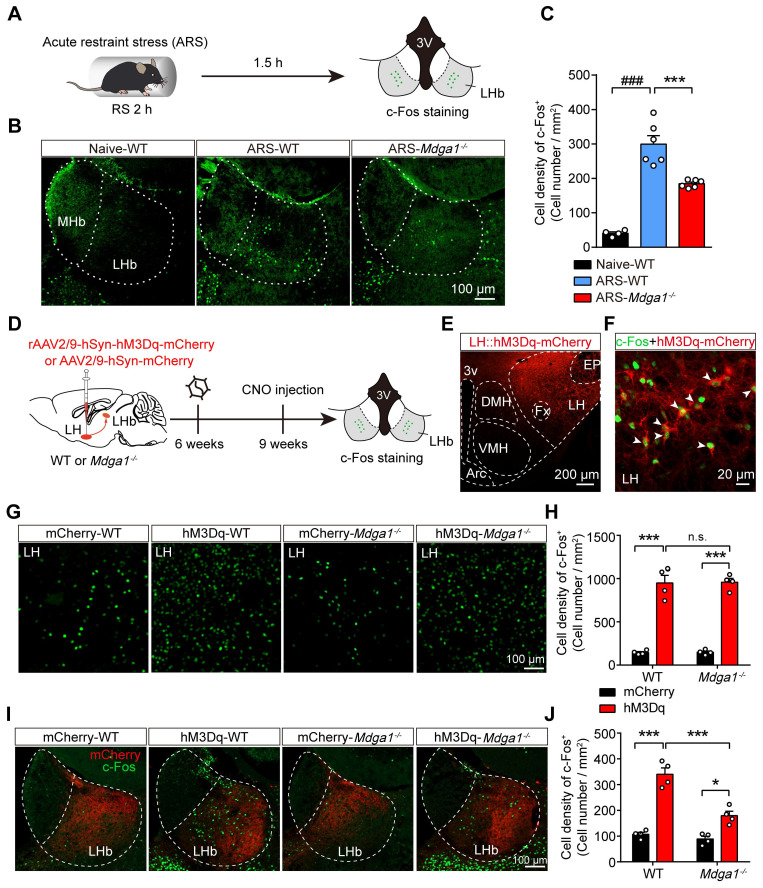
** Knockout of *Mdga1* confers resistance to ARS-induced activation of the LHb and the chemogenetic activation of LH-LHb pathway. (A)** Schematic diagram of the experimental procedure is shown. **(B)** Representative images of c-Fos immunoreactivity in each group. **(C)** The density of c-Fos-positive cells in the LHb of WT was significantly increased after exposure to ARS when comparing naive animals, and the *Mdga1* KO was resistant to the ARS-induce activation of LHb. n = 3 mice for Naive, n = 6 mice for ARS-WT, n = 6 mice for ARS-*Mdga1^-/-^*. **(D)** Schematic diagram of the experimental procedure. **(E)** Confocal image showing hM3Dq-mCherry expression in the LH of a WT animal. **(F)** Merged image showing colocalization of c-Fos and hM3Dq-mCherry expression as indicated by white arrows. **(G)** Representative images of c-Fos immunoreactivity in each group. **(H)** CNO administration in LH::hM3Dq mice significantly increased c-Fos expression in the LH compared to controls, and c-Fos expression in the LH did not differ between *Mdga1^-/-^*mice and WT mice. n = 4 mice for each group. **(I)** Representative images of c-Fos immunoreactivity in each group. **(J)** CNO administration in LH::hM3Dq mice significantly increased c-Fos expression in the LHb compared to controls, and the *Mdga1* KO was resistant to the CNO-induce activation of LHb. n = 4 mice for each group. Data are presented as the mean ± SEM. n.s., no significant difference; **p < 0.01, ***p < 0.001, ##*p* < 0.01, ###*p* < 0.001, one-way ANOVA with Tukey's multiple comparisons test for **(C)**, two-way ANOVA with Bonferroni's multiple comparisons test for **(H)** and **(J)**.

Previous studies revealed that the stress-induced hyperactivation of LHb neurons is involved in the onset of depression 15, 50. We further explored whether knocking out *Mdga1* inhibited stress-induced activation of LHb neurons. We used c-Fos signals as a proxy for neural activity. *Mdga1^-/-^* mice and WT littermates were subjected to acute restraint stress (2 h ARS) or remained undisturbed in their home cage before sacrifice (Figure 5A). Post-staining for c-Fos signals demonstrated that ARS activated the LHb neurons of WT mice compared with their home cage controls (Figure 5B, C). However, the density of c-Fos positive neurons of LHb from *Mdga1^-/-^* restraint stress mice was significantly lower than that of WT mice (Figure 5B, C). Thus, these results suggest that deficiency of MDGA1 significantly alleviates ARS-induced activation of LHb neurons.

Given that the lateral hypothalamus (LH) provides physiologically relevant input to the LHb during stress 15, 51, and LH-LHb synaptic potentiation is determinant in stress-induced depression 15, 50, we next examined whether chemogenetic activation of LH-induced LHb neuron hyperactivation was attenuated by MDGA1 deficiency. The Gq-coupled excitatory designer receptor exclusively activated by designer drugs (DREADD)-hM3Dq, was introduced to the LH of WT and *Mdga1^-/-^* mice by viral expression of rAAV2/9-hSyn-hM3Dq-mCherry (Figure 5D, E). To verify the CNO-mediated activation in LH::hM3Dq neurons, we performed whole-cell recordings in brain slices after 3 weeks of viral expression, and found that the spontaneous firing rate of LH::hM3Dq neurons was significantly increased upon CNO (5 μM) application (Figure S3A-C). Additionally, we found that the *in vivo* stimulation of LH::hM3Dq neurons in WT mice (hM3Dq-WT) and *Mdga1^-/-^* mice (hM3Dq-*Mdga1^-/-^*), *via* systemic CNO administration, enhanced the expression of c-Fos in the LH, compared to that in control mice (mCherry-WT) and *Mdga1^-/-^* mice (mCherry-*Mdga1^-/-^)* expressing rAAV2/9-hSyn-mCherry (Figure 5F-H). Moreover, we found that chemogenetic activation of LH-induced LHb neuron hyperactivation was attenuated by MDGA1 deficiency (Figure 5I, J). This was not due to differences in LH activation, as WT and *Mdga1^-/-^* mice showed comparable c-Fos expression in the LH following the CNO administration (Figure 5G, H). Taken together, these results indicate that MDGA1 deficiency attenuated the hyperactivation of LHb neurons following the chemogenetic activation of the LH.

### Conditional *Mdga1* knockout in the LHb does not alter depressive-like behaviors but prevents CRS-induced depression onset in mice

**Figure 6:**
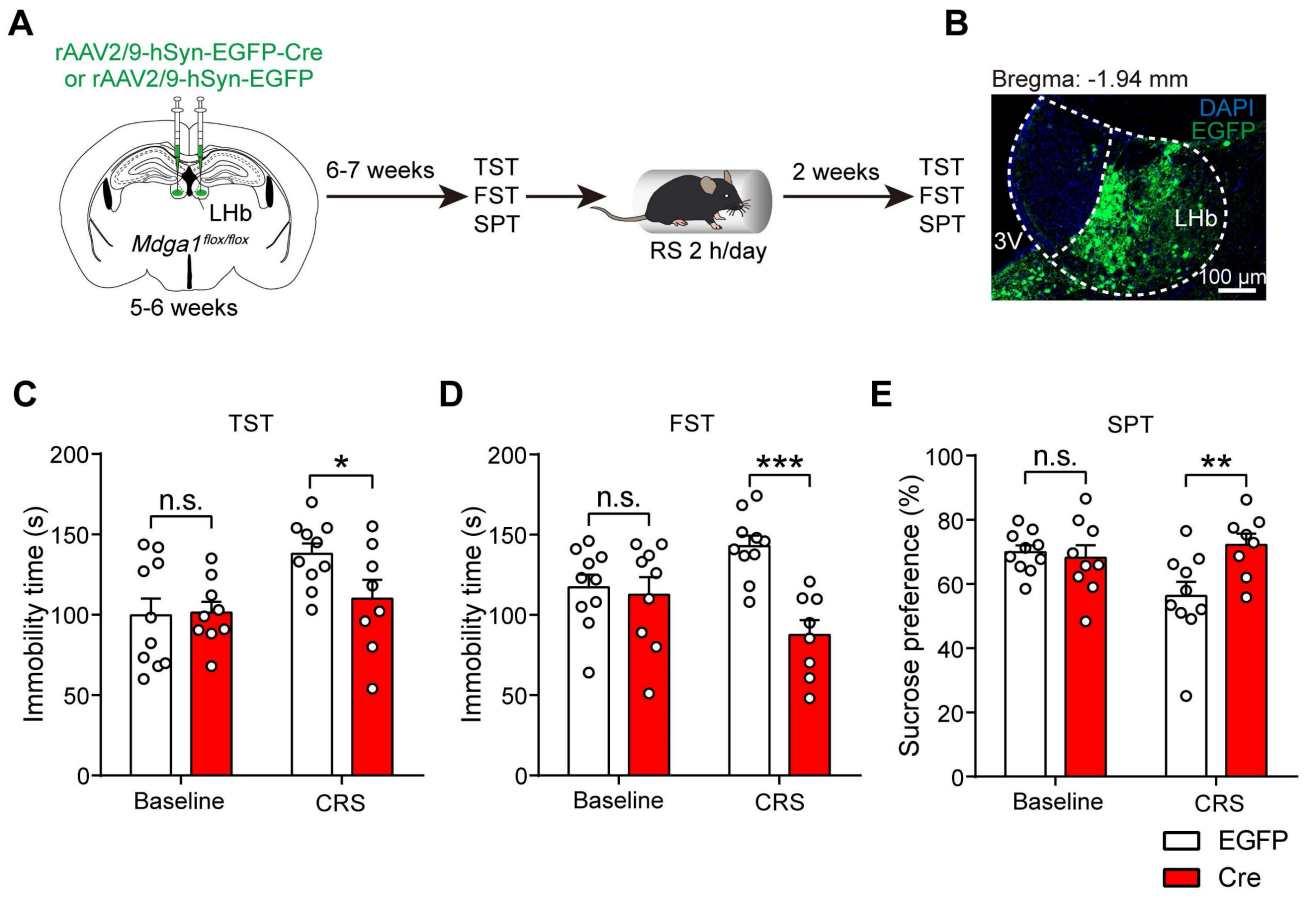
** Conditional *Mdga1* knockout in the LHb does not alter depressive-like behaviors but prevents CRS-induced depression onset. (A)** Schematic diagram of the experimental procedure. **(B)** Representative images show the rAAVs injection site in the LHb. **(C)** Deletion of *Mdga1* in the LHb did not alter immobility time of unstress mice but suppressed immobility of CRS mice in the TST. **(D)** Deletion of *Mdga1* in the LHb did not alter immobility time of unstress mice but suppressed immobility of CRS mice in the FST. **(E)** Deletion of *Mdga1* in the LHb did not alter sucrose preference of unstress mice but increased sucrose preference of CRS mice in the SPT. Baseline: n = 10 mice for EGFP, n = 9 mice for Cre; CRS: n = 10 mice for EGFP, n = 8 mice for Cre. Data are presented as mean ± SEM. n.s., no significant difference; *p < 0.05, **p < 0.01, two-way ANOVA with Bonferroni's multiple comparisons test.

Given that the hyperactivity of LHb neurons contributes to the pathophysiology of depression 7, and *Mdga1* deficiency attenuated the activity of LHb neurons, we next examined whether conditional knockout of *Mdga1* in the LHb altered rodent behavioral analogs of anhedonia and behavioral despair. The bilateral LHb of *Mdga1*^flox/flox^ mice were injected with rAAV2/9-hSyn-EGFP-Cre or rAAV2/9-hSyn-EGFP, followed by a 6-7 weeks recovery period before mice were subjected to depressive-like behavioral tests (Figure 6A, B). We found that conditional knockout of *Mdga1* in the LHb did not result in depressive-like behaviors (Figure 6C-E). However, *Mdga1*-cKO mice demonstrated lower stress-induced depressive-like behaviors in the tail suspension test (TST), forced swim test (FST), and sucrose preference test (SPT) following CRS (Figure 6C-E). These results indicate that conditional knockout of *Mdga1* in the LHb prevented the CRS-induced depression onset, specifically in tests for behavioral despair (TST, FST) and anhedonia (SPT).

### Disruption of MDGA1/Nlgn2 complex binding enhances inhibitory synaptic transmission in the LHb

**Figure 7:**
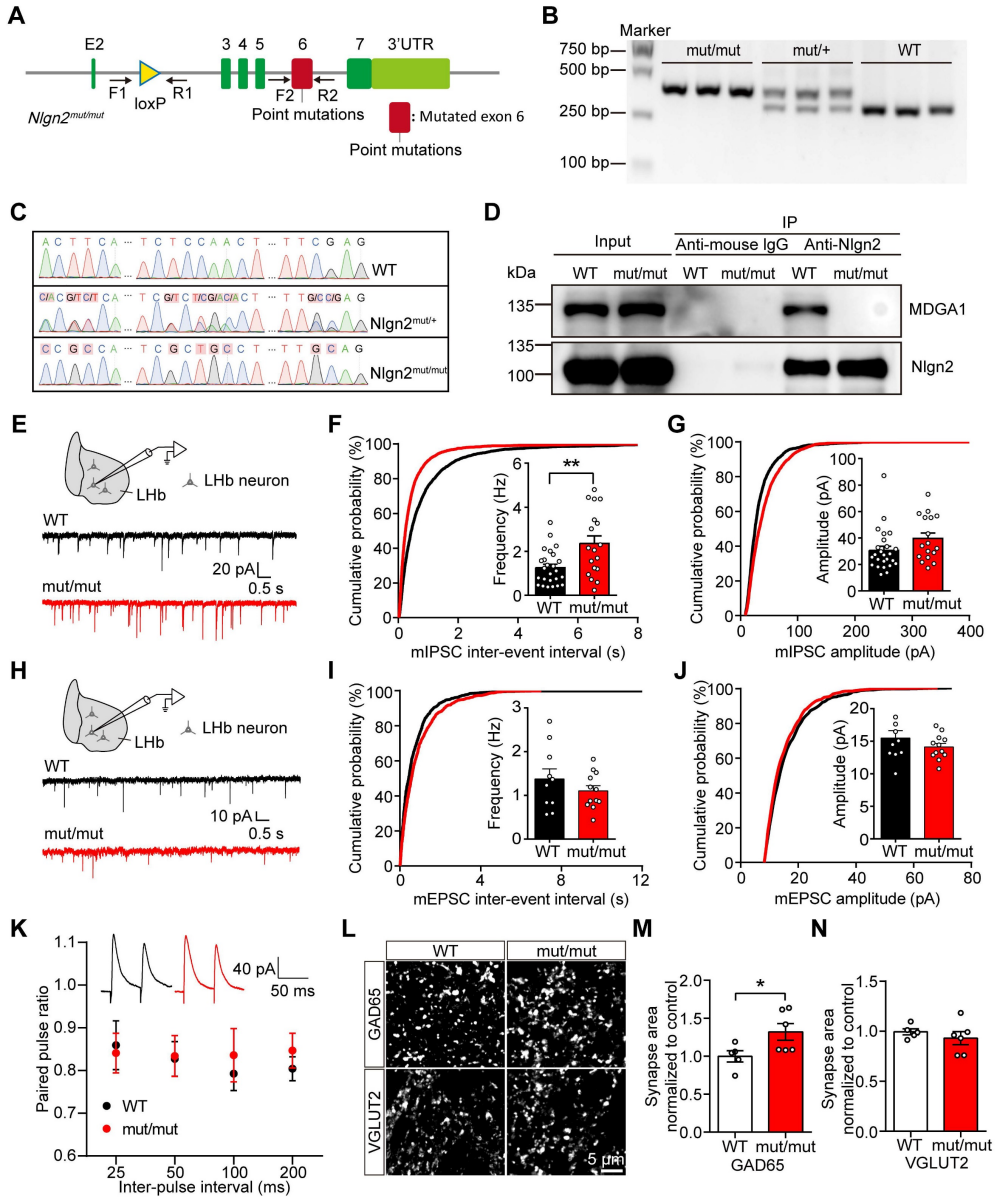
** Preventing MDGA1/Nlgn2 interaction through *Nlgn2* point mutation increases mIPSCs frequency and inhibitory synaptic density in the LHb. (A)** Schematic diagram of *Nlgn2* gene mutation.** (B)** PCR genotyping of genomic DNA from *Nlgn2^mut/mut^*, *Nlgn2^mut/+^* and WT mice using forward and reverse primers (F1, R1) revealed a WT band at 290 bp and a flox-inserted band at 354 bp. **(C)** Sanger sequencing results confirm the *Nlgn2* mutations on genomic DNA from the homozygous, heterozygous, and WT mice. **(D)** Immunoprecipitation results show that MDGA1 does not bind to Nlgn2 in *Nlgn2^mut/mut^* mice. **(E)** Schematic diagram of recording (top) and representative mIPSCs traces (bottom). **(F)** *Nlgn2* mutant neurons exhibited a significant increase in mIPSC frequency relative to WT neurons. n = 25 neurons, from 3 mice for WT, n = 18 neurons, from 4 mice for Mutant.** (G)** mIPSC amplitude did not differ between the *Nlgn2* mutant and WT groups. n = 25 neurons, from 3 mice for WT, n = 18 neurons, from 4 mice for Mutant. **(H)** Schematic diagram of recording procedures (top) and representative mIPSCs traces (bottom). **(I)** mEPSC frequency did not differ between the WT and *Nlgn2* mutant groups. n = 10 neurons, from 3 mice for WT, n = 12 neurons, from 3 mice for Mutant.** (J)** mEPSC amplitude did not differ between the WT and Mutant groups. n = 10 neurons, from 3 mice for WT, n = 12 neurons, from 3 mice for Mutant.** (K)** Paired-pulse facilitation (PPF) was normal in the LHb of *Nlgn2* mutant mice. n = 14 neurons, from 3 mice for WT group; n = 16 neurons from 3 mice for the *Nlgn2* mutant group; *p* = 0.5712; F _(1, 112)_ = 0.3226; two-way ANOVA.** (L)** Representative confocal images from LHb of WT and *Nlgn2* mutant brain sections immunolabeled with inhibitory presynaptic marker GAD65 and excitatory presynaptic marker VGLUT2 are shown. **(M)** Quantification of punctate integrated intensity per tissue area showed a significant increase in GAD65 in LHb of *Nlgn2* mutant mice. n = 5 mice for WT, n = 6 mice for Mutant.** (N)** Quantification of punctate integrated intensity per tissue area showed no change in VGLUT2 in the LHb of Mutant mice. n = 5 mice for WT, n = 6 mice for Mutant. Data are presented as mean ± SEM. *p < 0.05, **p < 0.01, unpaired t test.

Studies of extracellular protein structures revealed a specific binding site, Site II on Nlgn2, that is required for interaction with MDGAs 47. In addition, Nlgn2 was shown to preferentially bind to MDGA1 in the mouse brain through a pull-down study 49. To further investigate whether the effect of MDGA1 loss is mediated through Nlgn2, we generated *Nlgn2^mut/mut^* transgenic mice (Figure 7A), which harbors mutations in the Site II that disrupt the interaction between Nlgn2 and MDGA1. The *Nlgn2* floxed alleles were validated by genotyping (Figure 7B). We confirmed the *Nlgn2* mutations at genomic and transcriptomic levels from the homozygous, heterozygous, and wild-type mice by sanger sequencing (Figure 7C). Using immunoprecipitation, we also confirmed that MDGA1 does not bind to Nlgn2 in *Nlgn2*^mut/mut^ mice (Figure 7D).

To evaluate the impact of *Nlgn2^mut/mut^* on the excitatory and inhibitory synaptic function of LHb neurons, the mIPSCs and mEPSCs of LHb neurons were recorded in acute brain slices from the WT littermate and *Nlgn2^mut/mut^* mice (Figure 7E-J). mIPSC frequency was increased in the LHb of *Nlgn2^mut/mut^* mice compared to control mice (Figure 7F), while the amplitude remained unchanged (Figure 7G). Additionally, mEPSC amplitude and frequency were comparable between the *Nlgn2^mut/mut^* mice and WT controls (Figure 7H-J). No differences were found in PPR of evoked IPSCs (Figure 7K), indicating the altered mIPSCs may be attributed to enhanced inhibitory synapse density. Therefore, we next performed an immunocytochemical analysis of GAD65 and VGLUT2. Quantitative imaging revealed that the punctate immunofluorescence of GAD65 was significantly increased, while quantitation of VGLUT2 revealed no change in excitatory presynaptic terminal density in the LHb from *Nlgn2^mut/mut^* mice relative to control mice (Figure 7L-M). Taken together, these findings are consistent with increased inhibitory synapse number and basal transmission in the LHb of *Nlgn2^mut/mut^* mice.

### Disrupting MDGA1/Nlgn2 interactions alleviates ARS- and chemogenetic LH activation-induced LHb neuron hyperactivation

**Figure 8:**
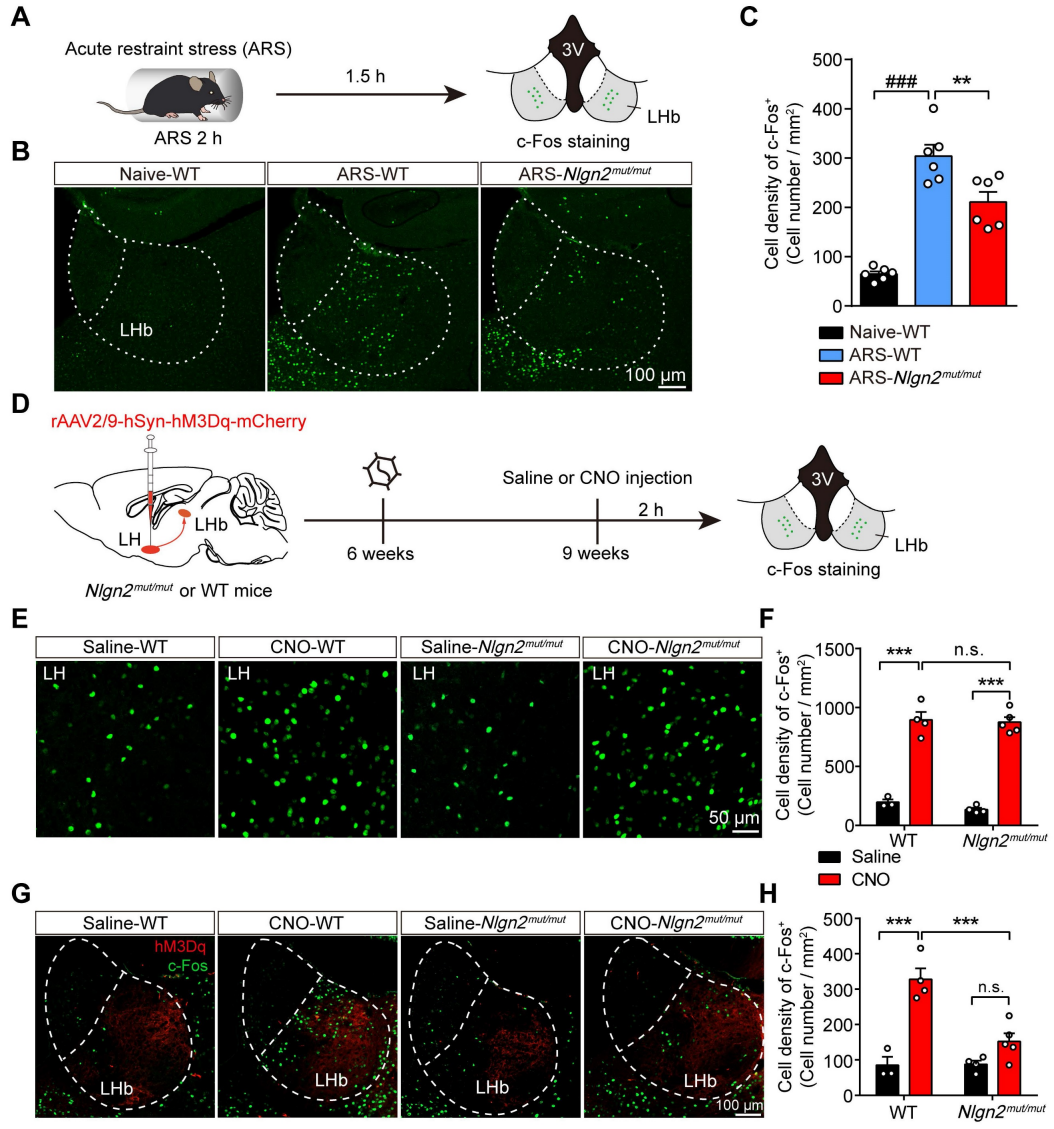
***Nlgn2^mut/mut^* confers resistance to ARS-induced activation of LHb and the chemogenetic activation of the LH-LHb pathway. (A)** Schematic diagram of the experimental procedure. **(B)** Representative images of c-Fos immunoreactivity in each group. **(C)** The density of c-Fos-positive cells in the LHb of WT animals were significantly increased after exposure to ARS when comparing naive animals, and *Nlgn2^mut/mut^* mice were resistant to the ARS-induce activation of LHb. n = 6 mice for Naive, n = 6 mice for ARS-WT, n = 6 mice for ARS-*Nlgn2^mut/mut^*. **(D)** Schematic diagram of the experimental procedure. **(E)** Representative images of c-Fos immunoreactivity in the LH of each group. **(F)** CNO administration in LH::hM3Dq mice significantly increased c-Fos expression in the LH compared to saline, and c-Fos expression in the LH did not differ between *Nlgn2^mut/mut^* mice and WT mice. n = 3 mice for Saline-WT, n = 4 mice for CNO-WT and Saline-*Nlgn2^mut/mut^*, n = 5 mice for CNO-*Nlgn2^mut/mut^*. **(G)** Representative images of c-Fos immunoreactivity in the LHb of each group. **(H)** CNO administration in WT LH::hM3Dq mice significantly increases c-Fos expression in the LHb compared to saline, whereas *Nlgn2^mut/mut^* mice were resistant to the CNO-induced activation of LHb. n = 3 mice for Saline-WT, n = 4 mice for CNO-WT and Saline-*Nlgn2^mut/mut^*, n = 5 mice for CNO-*Nlgn2^mut/mut^*. Data are presented as the mean ± SEM. n.s., no significant difference; *p < 0.05, **p < 0.01, ##*p* < 0.01, ###*p* < 0.001, one-way ANOVA with Tukey's multiple comparisons test for **(C)**, two-way ANOVA with Bonferroni's multiple comparisons test for **(F)** and **(H)**.

Since preventing MDGA1/Nlgn2 binding in *Nlgn2^mut/mut^* mice enhanced inhibitory synaptic transmission in LHb neurons, we next explored whether the *Nlgn2* mutation also alleviated ARS-induce activation of LHb neurons and chemogenetic activation of the LH-LHb pathway. To test this, *Nlgn2^mut/mut^* mice and WT mice were subjected to acute restraint stress (2 h ARS) or remained undisturbed in their home cage before sacrifice (Figure 8A). Post-staining for c-Fos signals following these treatments demonstrated that ARS activated the LHb neurons of WT mice compared with their home cage controls (Figure 8B, C). However, the density of c-Fos-positive neurons of LHb from *Nlgn2^mut/mut^* mice exposed to ARS were significantly lower than that of WT littermates (Figure 8B, C). Thus, these results indicate that disruption of MDGA1/Nlgn2 interactions alleviates LHb neuron activation following ARS.

We next examined whether *Nlgn2^mut/mut^* alleviates exaggerated neuronal activity following chemogenetic activation of the LH-LHb pathway. rAAV2/9-hSyn-hM3Dq-mCherry virus was injected into the LH of WT and *Nlgn2^mut/mut^* mice, and the activation of the LH-LHb pathway was assessed by immunofluorescence staining for c-Fos following saline or CNO administration (Figure 8D). Chemogenetic activation of LH neurons enhanced the activity of LHb in the WT group compared to the control mice (Figure 8E-H). Interestingly, chemogenetic activation of LH-induced LHb neuron hyperactivation was attenuated by disruption of MDGA1/Nlgn2 interactions (Figure 8G, H). This was not due to differences in LH activation as WT and *Nlgn2^mut/mut^* mice showed comparable c-Fos expression in the LH following the CNO administration (Figure 8E, F). These results indicate that preventing MDGA-Nlgn2 binding attenuates hyperexcitation within the LHb following chemogenetic activation of the LH-LHb pathway.

### Preventing MDGA1-Nlgn2 interactions in the LHb alleviates chronic stress-mediated depression-like behaviors in mice

**Figure 9:**
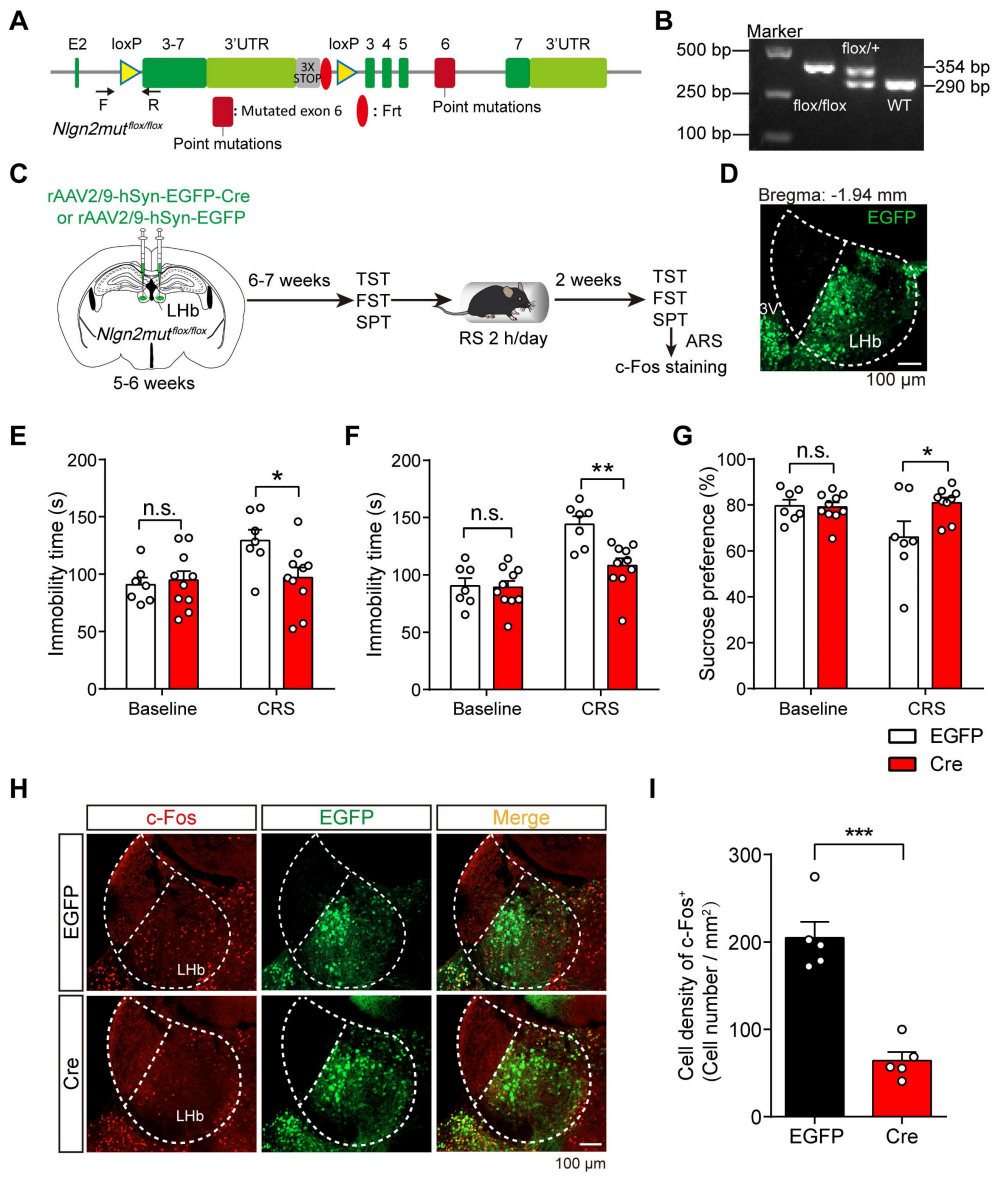
** Conditional knock-in of the *Nlgn2* mutation in the LHb selectively reduces chronic stress-induced depressive-like behaviors. (A)** Gene targeting strategy for *Nlgn2mut^flox/flox^* mice. **(B)** PCR genotyping of genomic DNA from *Nlgn2mut^flox/flox^*, *Nlgn2mut^flox/+^* and WT mice. **(C)** Schematic diagram of the experimental procedure is shown. **(D)** Representative images show the rAAV injection site in the LHb. **(E)** *Nlgn2* mutation in the LHb did not alter immobility time of unstress mice but suppressed immobility of CRS mice in the TST. n = 7 mice for EGFP, n = 10 mice for Cre. **(F)** *Nlgn2* mutation in the LHb does not alter immobility time of unstress mice but suppressed immobility of CRS mice in the FST. n = 7 mice for EGFP, n = 10 mice for Cre. **(G)** *Nlgn2* mutation in the LHb did not alter sucrose preference of unstress mice but increased sucrose preference of CRS mice in the SPT. Baseline: n = 7 mice for EGFP, n = 10 mice for Cre; CRS: n = 7 mice for EGFP, n = 9 mice for Cre. **(H)** Representative images of c-Fos immunoreactivity in the LHb of each group. **(I)** *Nlgn2* mutation in the LHb decreased the density of c-Fos-positive cells after ARS. n = 5 mice for EGFP, n = 5 mice for Cre. Data are presented as the mean ± SEM. n.s., no significant difference; *p < 0.05, **p < 0.01, ***p < 0.001, two-way ANOVA with Bonferroni's multiple comparisons test for **(E)**-**(G)**, unpaired t test for **(I)**.

To probe whether the disruption of MDGA1 and Nlgn2 interactions in the LHb also alleviates chronic stress-mediated depression-related behaviors in mice, we generated transgenic mice with a conditional knock-in *Nlgn2* mutation (*Nlgn2mut^flox/flox^*) (Figure 9A, B). To selectively manipulate *Nlgn2* mutation in the LHb, we stereotaxically targeted the LHb bilaterally with rAAV2/9-hSyn-EGFP-Cre (Cre) or rAAV2/9-hSyn-EGFP (EGFP) as a control in *Nlgn2mut^flox/flox^* mice at the age of 5-6 weeks (Figure 9C, D). Mice were then subjected to a series of depressive-like behavioral tests after 6-7 weeks of viral expression. We found that disruption MDGA1/Nlgn2 interactions in LHb did not alter baseline depressive-like behaviors (Figure 9E-G). Interestingly, we found that binding-deficient *Nlgn2* mutations restricted to the LHb prevented the onset of depression in the Cre group following CRS, while the EGFP control mice exhibited depressive-like behaviors assessed by the TST, FST, and SPT (Figure 9E-G). In addition, the density of c-Fos-positive neurons in the LHb from the Cre group were significantly lower than that of EGFP group following ARS (Figure 9H, I). Taken together, these results indicate that disruption of the interaction between MDGA1 and Nlgn2 in the LHb alleviates chronic stress-mediated depressive-like behaviors in mice through suppression of LHb activation during CRS.

## Discussion

In this study, we showed that MDGA1 and Nlgn2 are highly expressed and co-localized within the LHb. Strikingly, this colocalization was enhanced following 2 weeks of chronic restraint stress (CRS), which also significantly decreased the mIPSC frequency of LHb neurons, suggesting hyperactivity within the LHb results from increased MDGA1/Nlgn2 complex formation, and subsequent downregulation of inhibition within the LHb. Accordingly, preventing MDGA1/Nlgn2 complex formation restored stress resilience in mice, as knocking out MDGA1 or introduction of an MDGA1 binding deficient variant of Nlgn2 increased both mIPSC frequency and GAD65 puncta within the LHb, which enhanced GABAergic synapse formation in the LHb and restored resistance to chronic stress-induced depression.

Functional imaging studies consistently reported hyperactivity within the LHb in patients with major depression 9, 10, and deep brain stimulation in this region relieved depression symptomology in humans 52, 53 and rat models 54, 55. Interestingly, ketamine a strong anesthetic that has profound single-dose effects in alleviating depression 56, reduced burst firing within the LHb in depression models 57, 58. Increased neuronal activity within the LHb indirectly suppresses neuromodulatory pathways, including dopaminergic, serotonergic and noradrenergic systems, through glutamatergic projections onto rostromedial tegmental nucleus (RMTg) neurons that suppress aminergic neurons 7, 8. This pathway (LHb-RMTg) provides a causal link between LHb hyperactivity and downregulation of mood regulation (serotonin), reward salience (dopamine) and arousal circuits (noradrenaline) throughout the brain. These neuromodulatory systems are generally downregulated in depression, and mediate specific aspects of major depression symptomology, including anhedonia (dopamine) 59 and learned helplessness (serotonin) 60.

Despite these insights, the precise molecular events that determine changes in LHb neuronal activity, synaptic function, and transmission are yet to be fully elucidated. Altered GABAergic function has been established in the LHb following repeated stress. Protein phosphatase 2A activity increases in the LHb following stress, which negatively regulates GABA_B_ receptor-mediated currents 61. These findings indicate that dis-inhibition of pyramidal neurons within the LHb promotes the onset of depression like-behaviors. Our data verify that upregulation of GABAergic synapses is sufficient for anti-depression symptomology in mice, consistent with diminished E/I balance within the LHb as pathogenic in stress-induced depression.

Nlgn2 is a potent synaptogenic protein at GABAergic synapses 35, 36 which is negatively regulated by the synapse repressor, MDGA1 40, 43, 46. Interestingly, MDGA1 SNPs were identified as a risk factor for bipolar disorder and schizophrenia 62, 63 and RNA-Seq analysis of 78 patients with major depressive disorder identified MDGA1 as a leading candidate gene for major depressive disorder 64. We found that MDGA1 and Nlgn2 express within the LHb where immunolabeling revealed co-localized MDGA1/Nlgn2. MDGA1 levels and colocalization with Nlgn2 were enhanced following 2 weeks of CRS and CRS significantly decreased the mIPSC frequency of LHb neurons, suggesting that chronic stress induces MDGA1/Nlgn2 complex formation which downregulates GABAergic synapses within the LHb. A recent study demonstrated that chronic stress similarly reduces the frequency of inhibitory synaptic transmission in CA1 pyramidal neurons by inhibiting the Nlgn2-MyosinVa (MyoVa) interaction in the hippocampus 65. Although a decrease in inhibitory synaptic transmission frequency induced by chronic stress has been observed in different brain regions, the underlying mechanisms are distinct. This highlights diverse intra- and extracellular mechanisms for the negative regulation of Nlgn2-mediated inhibitory synaptic function, indicating the critical role of Nlgn2 in chronic stress-induced neurological disorders. These findings provide molecular targets and diverse intervention strategies for disease treatment. Consistent with a role in repressing LHb GABAergic synapses, inhibitory synaptic transmission was increased within this region following genetic reduction of MDGA1. Critically, the frequency but not amplitude of mIPSCs was increased, indicating that GABAergic synapse number but not basal strength was elevated in *Mdga1^-/-^* mice. These results were corroborated by an increase in GAD65^+^ puncta, whereas no changes in functional assessment or immunostaining results for glutamatergic synapses were observed.

Conditional knockout of *Mdga1* in the LHb confirmed a selective enhancement in mIPSC frequency and GAD65^+^ puncta, whereas glutamatergic synapse properties remained unaltered. This data indicates that MDGA1/Nlgn2 interactions maintain E/I balance within the LHb well into adulthood, suggesting that altering the levels of these organizers or their interactions could set the conditions for resistance or susceptibility to major depression. Accordingly, by biasing LHb output gain in favor of excitation, MDGA1/Nlgn2 complex formation would act as a molecular actuator for depression circuits. Notably, MDGA1 co-expression with Nlgn2 was not observed in the LH, suggesting that the effects of this complex are restricted to the LHb, consistent with a specific role in encoding the negative valence of stress-induced depression and not more generalized forms of depression.

Increased neuronal activity is a hallmark of the stress response with the LHb 8. Accordingly, shunting excitation within the LHb should limit the feedforward excitatory inputs to other brain regions, notably the RMTg, that mediate the stress response. Consistent with this hypothesis, upregulation of GABAergic inputs following genetic reduction of MDGA1, prevented the ARS-induced neuronal hyperactivity within this circuit, most likely through liberation of Nlgn2. Therefore, genetic removal of MDGA1 prevents the LHb from signaling the onset of depression during stressful events, providing a novel therapeutic target for limiting the effects of stress on genesis of depressive states.

Further testing of the prophylactic effects of MDGA1 manipulation showed that increasing inhibition within the LHb was sufficient for short-circuiting depression networks. Specifically, we found that chemogenetic activation of the LH, which provides the primary inputs that increase neural activity within the LHb 7, 15, 51, was sufficient for limiting excitatory inputs converging on LHb neurons. In addition to providing evidence for a critical role for MDGA1-Nlgn2 interactions in regulating synaptic responses to stress, these findings indicate that reducing MDGA1/Nlgn2 complex formation within the LHb is sufficient for preventing the transfer of elevated LH neuronal activity to LHb neurons.

Importantly, *Mdga1* knockout did not alter the propensity for onset of depression *per se*; However, depression resistance was consistently observed when mice underwent behavioral tests for exaggerated stress responses (TST, FST, SPT). The tail suspension and forced swim tests both measure the tendency towards behavioral despair, a rodent analog for increased propensity towards hopelessness observed in some depressed patients 66. Alternatively, the sucrose preference test provides measures of preference for rewarding stimuli which when decreased, is consistent with anhedonia (impaired pleasure pursuit) 67. cKO of *Mdga1* reversed measures of both behavioral despair and anhedonia in mice experiencing CRS. This result links MDGA1/Nlgn2 interactions to canonical features of depression symptomology, revealing hitherto unknown molecular interactions that constitute part of the cellular basis for stress-induced synaptic changes underlying despair and anhedonia in a rodent model.

ARS was sufficient for increasing neuronal activity within the LHb, as demonstrated by increased c-Fos reactivity following 2 h of ARS. However, the c-Fos expression in *Mdga1^-/-^* mice subjected to restraint stress was significantly reduced compared to that in WT mice. This finding indicates that reducing MDGA1 halts the onset of increased neural activity within the LHb in response to acute as well as chronic stressors. Notably, these effects are observed during ARS when the cellular events that prime depression onset take place, suggesting that MDGA1 modulates stress resistance at the earliest stages of stress-induced depression.

*Mdga1* knockout also prevented the upregulation of LHb neural activity in response to chemogenetic stimulation of the LH, providing further evidence that removal of MDGA1 constraint of Nlgn2 is sufficient for shunting the stress-to-depression axis across several paradigms that induce hyperactivity within the LHb. Use of an MDGA1 binding deficient Nlgn2 genetic variant further confirmed the central regulatory role this complex (MDGA1/Nlgn2) plays in providing the molecular substrates for stress-mediated depression resilience. Similar to *Mdga1^-/-^* mice, *Nlgn2^mut/mut^* mouse slices harbored pyramidal neurons with increased mIPSC frequencies and GAD65 puncta density, indicating more GABAergic synapses. LHb neuronal responses to chemogenetic activation of the LH-LHb pathway or acute restraint stress were similarly muted in the *Nlgn2^mut/mut^* mice, confirming that decreased binding of MDGA1 to Nlgn2 is specifically required for conferring resistance to stress-induced depression.

Behavioral measures of depression in Nlgn2 mutants revealed a marked reduction in depression indicators following CRS. Mice with Nlgn2 mutation in the LHb showed reduced behavioral despair following CRS, as demonstrated by sustained motoric responses in the tail suspension and forced swim tests (Figure 9). Similarly, neurobehavioral indicators for anhedonia were diminished, as shown through increased sucrose preference. Collectively, these results indicate that the onset and symptomology of stress-induced depression are heavily contingent upon direct interactions between MDGA1 and Nlgn2 within the LHb, such that preventing complex formation reduces the negative impacts of acute and chronic restraint stress on subsequent measures of depression. Although hevin and MDGAs, which theoretically exert opposite actions, occupy overlapping binding sites on Nlgn2 in in vitro studies 68, the *in vivo* effects of hevin on Nlgns-Nrxns are still controversial 69, 70. Moreover, the enhanced inhibitory synaptic transmission in the LHb of *Nlgn2^mut/mut^* mice indicate disrupting MDGA1/Nlgn2 interactions plays a dominant role.

Further studies are needed to elucidate how MDGA1 expression becomes elevated (and whether this takes place at translational and/or transcriptional levels), and if so how MDGA1 is stabilized at synapses. Additional probing is required to further reveal how stress exposure couples to mechanisms that govern binding/unbinding of the MDGA1-Nlgn2 complex. Single molecule tracking established that MDGAs show high diffusion rates at synapses, in tandem with limited synapse “coverage”, suggesting that MDGA-Nlgn interactions occur at the synapse periphery 71. This supports a model in which MDGAs may confine Nlgns outside of synapses, which may be reversed upon synaptic activation, freeing up Nlgns for synaptic changes associated with negative experiences or stress-based insults 71. The effects of neuronal MDGA deletions in dissociated cultured hippocampal neurons showed that MDGAs perform activity-dependent synapse type-specific suppression, with MDGA1 suppressing the density, transmission, and strength of GABAergic synapses 72, supporting a model in which increased colocalization of MDGA1/Nlgn2 suppresses GABAergic synapses following stress. Interestingly, neuroligin synaptic localization is determined in part by posttranslational modifications, including tyrosine phosphorylation 73. Testing if LH-induced hyperexcitability within the LHb promotes posttranslational modifications of neuroligins that reduces synaptic confinement and thereby promote MDGA1-Nlgn2 complex formation at the synapse periphery would be important for addressing this question.

Likewise, it is important to determine the network level effects of MDGA1-Nlgn2 complex formation. Focusing on synaptic plasticity would likely reveal important circuit-level effects, particularly in light of the enhanced LTP following stress within the LHb 15, 50. Previous findings demonstrated impaired LTP within the hippocampus of *Mdga1^-/-^* mice that was rescued by inhibition of GABA_A_ receptors 20, 40. It will be interesting to test if deletion of MDGA1 similarly prevents LH-LHb potentiation following stress, which would be the predicted outcome under conditions of increased inhibition.

Finally, manipulating the expression MDGA1 or binding of MDGA1 to Nlgn2 can serve as a molecular tool for selectively modulating inhibitory synaptic drive in the LHb to prevent the onset of major depression following stress. Elevated levels of MDGA1 within the LHb, further emphasizes the highly regional specificity of MDGA1 expression in the brain, which may add to the complexity of Nlgn2's regulation of mood related brain circuitry as well as provide specific drug target for depression. Our results indicate that genetic reduction of MDGA1 rendered LHb neurons less prone to overexcitation, an effect that was reversed following MDGA1 reconstitution in *Mdga1^-/-^* neurons. Introduction of an MDGA1-binding deficient version of Nlgn2 provided strong supporting evidence that the LHb neuron modulating effects of stress were contingent upon MDGA1-Nlgn2 interactions. Accordingly, identifying interfering peptides or drugs that liberate Nlgn2 from MDGA1 suppression may hold promise for restoring synaptic balance within the LHb thereby attenuating the downstream engagement of depression circuitry.

## Materials and Methods

### Animals

*Mdga1^-/-^* mice, designed as a knockout-first allele approach 74, were generated using the ES cells transduced with the targeting plasmid (*Mdga1^tm1a(EUCOMM)Hmgu^*) purchased from the European Conditional Mouse Mutagenesis Program (EUCOMM). The mice were continually bred on a C57BL/6J background. The *Mdga1^flox/flox^* mice were obtained by crossing *Mdga1^-/-^* mice with FlpO (FLP) recombinase-expressing mice (Rosa26-FlpO, B6.129S4-Gt (ROSA) 26Sor^tm1(FLP1)Dym^/RainJ, stock number: 009086) purchased from Biocytogen Pharmaceuticals Co., Ltd (China). The *Nlgn2mut^flox/flox^* mice were generated by targeting a mouse ES line in collaboration with Biocytogen Pharmaceuticals Co., Ltd (Beijing, China). The* Nlgn2^mut/mut^* mice obtained by crossing *Nlgn2mut^flox/flox^* mice with CMV-Cre mice (B6.C-Tg (CMV-cre)1Cgn/J, strain #006054, The Jackson Laboratory). Mice were housed in a room at a 12-h light/dark cycle with access to food and water *ad libitum* under specific pathogen-free conditions. All the mice used were male (8-12 weeks) unless otherwise specified. Mice were 5-6 weeks old at the time of injection and allowed to recover for 6-7 weeks prior to experiments. All procedures were conducted in accordance with the guidelines of Zhejiang University Animal Care and Use Committees.

### Immunofluorescence and imaging

Mice were deeply anesthetized with overdose 2% pentobarbital sodium and then intracardially perfused with 20 mL cold 0.01 mol/L phosphate-buffered saline (PBS) followed by 20 mL chilled 4% paraformaldehyde (PFA, P0099, Beyotime Biotechnology, China) in PBS (C0221A, Beyotime Biotechnology, China). The brains were removed and then post-fixed overnight at 4 °C in PFA. After that, the brains were subjected to gradient dehydration using PBS solution containing 15% and 30% sucrose, then were embedded in OCT (4583, SAKURA, Japan) and sectioned coronally (30 μm) with a freezing microtome (CryoStar NX70, Thermo Fisher Scientific, USA). For immunofluorescent staining, the sections were blocked with a blocking buffer (QuickBlock™, P0260, Beyotime Biotechnology, China) for 1 h at 37 °C and subsequently incubated with primary antibodies: anti-MDGA1 (1:500, rabbit, 421002, Synaptic systems, Germany), anti-beta Gal (1:400, chicken, ab9361, Abcam, USA), anti-NeuN (1:500, rabbit, PA5-78639, Invitrogen, USA), anti-CaMKII alpha (1:300, rabbit, ab5683, Abcam, USA), anti-parvalbumin (1:4,000, mouse/IgG1, P3088, Sigma-Aldrich, USA), anti-GAD65 (1:200, mouse/IgG2a, AB_528264, Developmental Studies Hybridoma Bank, USA), anti-VGLUT2 (1:500, rabbit, 135402, Synaptic systems, Germany), anti-c-Fos (1:500, guinea pig, 226308, Synaptic system, Germany), anti-Neuroligin2 (1:500, mouse/IgG1, 75-451, NeuroMab, USA) and anti-Gephyrin (1:500, mouse/IgG2b, 75-443, NeuroMab, USA). After washing with PBS, the sections were subsequently coupled with the corresponding fluorophore-conjugated secondary antibodies (1:1,000, Thermo Fisher, USA) for 2 h at 37 °C. Finally, the wahsed sections were coverslipped using Dapi Fluoromount-G (0100-20, SouthernBiotech, USA) for nuclear labeling. Fluorescence signals were visualized under a confocal microscope (SP8, Leica Microsystems, Germany) using the same parameters for each marker among sections. The quantification of excitatory (VGLUT2) and inhibitory (GAD65) synaptic markers was analyzed with ImageJ software as we described previously 75.

### Western blot

Tissues were homogenized in Syn-PER Reagent (87793, Thermo Scientific, USA) and PMSF (ST506, Beyotime Biotechnology, China) at a ratio of Syn-PER Reagent: PMSF = 100:1. Lysates were centrifuged at 1,200 g for 10 min at 4 °C to remove debris, to obtain homogenates. Then the samples were centrifuged at 15,000 g for 20 min at 4 °C to remove the supernatant and obtain the synaptosome pellet. Finally, Syn-PER Reagent was added to suspend the synaptosome pellet. The BCA protein assay kit (P0010S, Beyotime Biotechnology, China) was used to quantify the protein concentration. Protein (20 μg) was loaded onto 8% SDS-PAGE gels for electrophoretic separation and transferred onto PVDF membranes (IPVH00010, Millipore, USA). The PVDF membranes were then blocked with 5% BSA in TBST buffer for 1 h at room temperature (RT) and were subsequently incubated with primary antibodies at 4 °C. The primary antibodies were as follows: anti-β-actin (1:5,000, mouse, #3700, Cell Signaling Technology, USA), anti-β-actin (1:5,000, rabbit, #4970, Cell Signaling Technology, USA), anti-MDGA1 (1:2,000, rabbit, #421002, Synaptic Systems, Germany), anti-Nlgn2 (1:2,000, mouse, #129511, Synaptic Systems, Germany). Appropriate secondary antibodies were added for 1 h at RT. Immunoreactive bands were visualized using Odyssey CLx (LI-COR, USA), and data were quantified using Fiji/ImageJ (NIH, USA).

### Co-immunoprecipitation (Co-IP)

Hippocampal tissue was collected and placed in western and IP lysis buffer (P0013, Beyotime Biotedhnology, China) containing PMSF (ST506, Beyotime Biotedhnology, China) and a protease inhibitor cocktail (P1010, Beyotime Biotedhnology, China). The tissue was homogenized for 2 min at 4 °C. After homogenization, the lysate was incubated on ice for 30 min to ensure complete lysis. The mixture was then centrifuged at 12,000 rpm for 15 min at 4 °C, the supernatant was collected and divided into three portions: one portion was mixed with 5× loading buffer (P0015, Beyotime Biotedhnology, China) and denatured directly for the input group, the second portion was incubated with an anti-mouse IgG1 antibody (negative control group, SC-3877, Santa Cruz, USA), the third portion was incubated with an anti-Nlgn2 mouse IgG1 antibody (IP group, #129511, SySy, Germany). Both the negative control and IP group samples were incubated overnight on a shaker at 4 °C. The next day, 30 μL of protein A/G magnetic beads (P2108, Beyotime Biotedhnology, China) were added to the antigen-antibody complexes and incubated at room temperature on a shaker for 2 h. The beads were separated using a magnetic rack and the supernatant was discarded. The beads were washed three times with 200 μL PBS. Finally, the washed beads were mixed with 2× loading buffer and denatured. The samples were analyzed via SDS-PAGE and western blot using the appropriate primary antibodies.

### Quantitative reverse transcription PCR (qRT‑PCR)

Total RNA was extracted from the hippocampus using TRIzol reagent (Invitrogen) according to the manufacturer's instructions. Total RNA was quantified using a spectrophotometer (Nanodrop 2000, Thermo Fisher). All the samples presented 260/280 nm ratios between 1.8 and 2.0. Next, cDNA was synthesized using the PrimeScript™ RT reagent kit (Takara, Japan). Real-time quantitative PCR was then performed using TB Green® Premix Ex Taq™ (Tli RNase H Plus, Takara, Japan) on the Real-Time PCR Detection System (CFX Connect™, BIO-RAD, USA). The primer sequences of *Mdga1* and GAPDH were as follows: *Mdga1* (Forward 5'- AACGGTGCATCAGACAGTGA-3', Reverse 5'-TGGTGGCTGTGCAGTTGTAG -3'), GAPDH (Forward 5'-GGTGAAGGTCGGTGTGAACG-3', Reverse 5'-CTC GCTCCTGGAAGATGGTG-3'). The results were analyzed using the 2^(-ΔΔCt)^ method.

### Ex vivo slice electrophysiology recording

Electrophysiological slice recording was performed as described previously 75. Briefly, mice were anesthetized with isoflurane and the brains were quickly removed to ice-cold oxygenated (95%O_2_/5%CO_2_) cutting solution containing (in mM): 120 choline chloride, 2.5 KCl, 7 MgSO_4_, 0.5 CaCl_2_, 1.25 NaH_2_PO_4_, 26 NaHCO_3_, 25 glucose, 3 sodium pyruvate and 5 sodium ascorbate. Coronal brain slice containing lateral habenula (300 μm thickness) were sectioned with VT1200S Vibratome (Leica, Germany). Slices were recovered in cutting solution for 15 min at 34 °C, and followed in oxygenated artificial cerebrospinal fluid (ACSF) containing (in mmol/L): 124 NaCl, 2.5 KCl, 2 MgSO_4_, 2.5 CaCl_2_, 1.25 NaH_2_PO_4_, 22 NaHCO_3_, and 10 glucose for at least 1 h at 25 °C before recording.

Slice was transferred to the recording chamber that continuously perfused with oxygenated ACSF (~2 ml/min) at 32 ± 1 °C. Neurons in the LHb were visualized with infrared optics using an upright fixed microscope equipped with a 40× water-immersion lens (BX51WI, Olympus, Japan) and a highly sensitive CCD camera (IR-1000E, DAGE-MTI, USA). Patch pipettes that made from filamented borosilicate glass capillary tubes (inner diameter, 0.84 μm) were pulled by a vertical pipette puller (PC-100, Narishige, Japan) with a resistance of 3-5 MΩ.

For mEPSCs recording, pyramidal neurons were held at -70 mV in the presence of picrotoxin (PTX, 100 μM) and tetrodotoxin (TTX, 1 μM), with the Csmethanesulfonate pipette solution containing (in mM): 130 Csmethanesulfonate, 5 NaCl, 1 MgCl_2_, 10 HEPES, 0.2 EGTA, 2ATP-Mg, 0.1 GTP-Na, and 5 QX314. The pH was adjusted to 7.3-7.4 with CsOH and osmolarity was adjusted to 285 mOsm with sucrose.

For mIPSC recording, pyramidal neurons were held at -70 mV in the presence of cyanquixaline (CNQX, 20 μM), DL-2-amino-5-phosphonopentanoic acid (DL-AP5, 20 μM) and TTX (1 μM), with the pipette solution containing (in mM): 100 CsCl, 30 Csmethanesulfonate, 5 NaCl, 1 MgCl_2_, 10 HEPES, 0.2 EGTA, 2 ATP-Mg, and 0.1 GTP-Na. The pH was adjusted to 7.3-7.4 with CsOH and osmolarity was adjusted to 285 mOsm with sucrose.

For PPR of evoked IPSCs recording, pyramidal neurons were clamp at 0 mV in the presence of 20 μM CNQX and 20 μM DL-AP5, with the Cs-methanesulfonate pipette solution. A stimulating electrode was placed 50-100 μm around recording electrode. Paired stimuli were delivered with different inter-stimulus intervals (25, 50, 100 or 200 ms). The ratios were calculated as 2^nd^ EPSC/1^st^ EPSC.

Recordings were performed with MultiClamp 700B amplifier and 1550B digitizer (Molecular Devices, USA). Series resistance was below 20 MΩ and monitored throughout the experiments. If the series resistance changed >20% during recording, the data were excluded. Data were sampled at 10 kHz and filtered at 3 kHz.

### Stereotaxic surgery and virus injection

Five to six weeks old mice were used for stereotaxic injection. Briefly, mice were deeply anesthetized with 2% pentobarbital sodium and fixed in a stereotaxic frame (RWD, China). An incision was made in the midline of the scalp, and a craniotomy was performed above the lateral habenula or lateral hypothalamus. Virus were injected into LHb (ML, ±0.46 mm; AP, -1.76 mm; DV, -2.70 mm) or LH (ML, ±1.06 mm; AP, -0.60 mm; DV, -4.78 mm) according to experiments through a pulled-glass micropipette with a 10-20 μm tip diameter connected to a 10 μL microliter syringe (Hamilton Co., USA). AAV was delivered using a stereotactic injector (Pump 11 Elite, Harvard Apparatus, USA) at a rate of 40 nL/min. After injection, the micropipettes were left in place for an additional 10 min to allow for diffusion before the pipette was slowly withdrawn. For *Mdga1* cKO in the LHb, *Mdga1^flox/flox^* mice were bilaterally microinjected with rAAV2/9-hSyn-EGFP-Cre (3.38 × 10^12^ vg/mL, 1:20, 200 nL, PT-0156, BrainVTA, China) or the control virus: rAAV2/9-hSyn-EGFP (2.27 × 10^12^ vg/mL, 1:20, 200 nL, PT-0241, BrainVTA, China) into the LHb. For *Mdga1* conditional restoration in the LHb, *Mdga1^-/-^* mice were bilaterally microinjected with rAAV2/9-hSyn-EGFP-FLP (3.57 × 10^12^ vg/mL, 1:20, 200 nL, PT-0803, BrainVTA, China) or the control virus: rAAV2/9-hSyn-EGFP (2.27 × 10^12^ vg/mL, 1:20, 200 nL, PT-0241, BrainVTA, China) into the LHb. For chemogenetic activation of LH neurons, mice were bilaterally microinjected with rAAV2/9-hSyn-hM3Dq-mCherry (titer: 1.63 × 10^13^ vg/mL, 1:10, 100 nL, Taitool Bioscience, S0425-9) or rAAV2/9-hSyn-mCherry (titer: 1 × 10^13^ vg/ml, 1:10, 100 nL, Taitool Bioscience, S0238-9) into the LH. For Nlgn2 conditional mutant in the LHb, mice were bilaterally microinjected with rAAV2/9-hSyn-EGFP-Cre (3.38 × 10^12^ vg/mL, 1:20, 200 nL, PT-0156, BrainVTA, China) or rAAV2/9-hSyn-EGFP (2.27 × 10^12^ vg/mL, 1:20, 200 nL, PT-0241, BrainVTA, China) into the LHb.

### Restraint stress

Mice were placed in restraint tubes fitted to body size with ventilation holes for 2 h (from 10:00 to 12:00) per day for 1 day (acute restraint stress, ARS) or consecutive 14 days (chronic restraint stress, CRS). During the restraint period, control mice were allowed to freely move around the cage, but fasted for water and food.

### Chemogenetic manipulations

For in vivo chemogenetic activation experiments, LH::hM3Dq and LH::Control mice received i.p. injections of either CNO (1 mg/kg in 0.5% DMSO/saline) or vehicle (0.5% DMSO/saline) 2 h prior to sacrifice for c-Fos staining.

### Forced swim test (FST)

Animals were individually placed into a transparent plexiglass cylinder (diameter 10 cm, height 25 cm) containing water at 23-25 ℃ and swam for 6 min under normal light. Water depth was set to prevent animals from touching the bottom with their tails or hind limbs. Animal behaviors were videotaped from the side. Immobility was assigned when no additional activity was observed other than that required to keep the mouse heads above the water. The time that mice spent in immobility in the last 4 min was quantified offline manually by an observer blinded to animal treatment. Animals were never allowed to drown during the test.

### Tail suspension test (TST)

Mice were suspended by the tip of the tail using adhesive tape. One end of the tape was attached to a horizontal table that surfaces above 30 cm from the floor. Animal behaviors were videotaped for 6 min. The immobile time during the last 4 min was counted offline by an observer blinded to animal treatment. Mice were considered immobile when they were completely motionless or passive swaying.

### Sucrose preference test (SPT)

Animals were single housed and habituated with two bottles of water for 2 days, followed by two bottles of 1% sucrose for 2 days. Animals were then water deprived for 24 h and then exposed to one bottle of 1% sucrose and one bottle of water for 2 h in the dark phase. Bottle positions were switched after 1 h (for 2 h test). Total consumption of each fluid was measured and sucrose preference was defined as the average sucrose consumption ratio during the first and second hour.

### Statistical analysis

All data are presented as the mean ± SEM. The unpaired student's t-test was used to compare the two groups. For comparison among three or more groups, one-way ANOVA with post‑hoc Tukey's multiple comparisons test was used. For data with more than one independent variable, two-way ANOVA with post‑hoc Bonferroni's t-test was used. In all cases, *p* < 0.05 was considered to be statistically significant.

### Study approval

All experimental procedures described in this article were approved by the animal care committee of Zhejiang University (No. ZJU20220294).

## Supplementary Material

Supplementary figures.

## Abbreviations

ARS: acute restraint stress
CRS: chronic restraint stress
FST: forced swim test
LHb: lateral habenula
LH: lateral hypothalamus
LS: lateral septum
MDGAs: MAM domain-containing GPI anchors
MyoVa: MyosinVa
mPFC: medial prefrontal cortex
Nlgn2: neuroligin2
Nlgns: neuroligins
Nrxns: neurexins
neo: neomycin
PPR: paired pulse ratio
PV: parvalbumin
RMTg: rostromedial tegmental nucleus
SPT: sucrose preference test
TST: tail suspension test
VGLUT2: vesicular glutamate transporter 2
β-gal: β-galactosidase
