# Supplementary material for: Chronic stress induces depression through MDGA1-Neuroligin2 mediated suppression of inhibitory synapses in the lateral habenula
Source: Theranostics. 2025 Jan 2;15(5):1842–63. doi: 10.7150/thno.104282 (PMC11780514; doi:10.7150/thno.104282)
Supplement: Supplementary file 1 — Supplementary figures. [file thnov15p1842s1.pdf]

Supplementary Material

Chronic stress induces depression through MDGA1-Neuroligin2 mediated suppression of inhibitory synapses in the lateral habenula

Xuehui Wang[1,#], Hao Wei[1,#], Zhe Hu[1,#], Jie Jiang[1,2,#], Xinyan Dong[1], Jinpiao Zhu[1], Haiyan Chen[1], Nils Brose[3], Noa Lipstein[3,4], Tonghui Xu[5], Steven A. Connor[2,*], Daqing Ma[1,6,*], Yicheng Xie[1,*]

[1]Perioperative and Systems Medicine Laboratory, Department of Anesthesiology, Children's Hospital, Zhejiang University School of Medicine, National Clinical Research Center for Child Health, Hangzhou, 310052, Zhejiang Province, China.

[2]Department of Biology, York University, 4700 Keele Street, Toronto, ON, M3J 1P3, Canada.

[3]Department of Molecular Neurobiology, Max Planck Institute for Multidisciplinary Sciences, Göttingen, 37075, Germany.

[4]Department of Molecular Physiology and Cell Biology, Leibniz-Forschungsinstitut für Molekulare Pharmakologie and NeuroCure Excellence Cluster, Berlin, 13125, Germany.

[5]Department of Laboratory Animal Science, Fudan University, Shanghai, 200032, China.

[6]Division of Anesthetics, Pain Medicine & Intensive Care, Department of Surgery and Cancer, Faculty of Medicine, Imperial College London, Chelsea and Westminster Hospital, London SW10 9NH, United Kingdom.

[*]Corresponding authors: Yicheng Xie (ycxie@zju.edu.cn), Daqing Ma (d.ma@imperial.ac.u) and Steven A. Connor (saconnor@yorku.ca)

[#]These authors equally contributed to this work

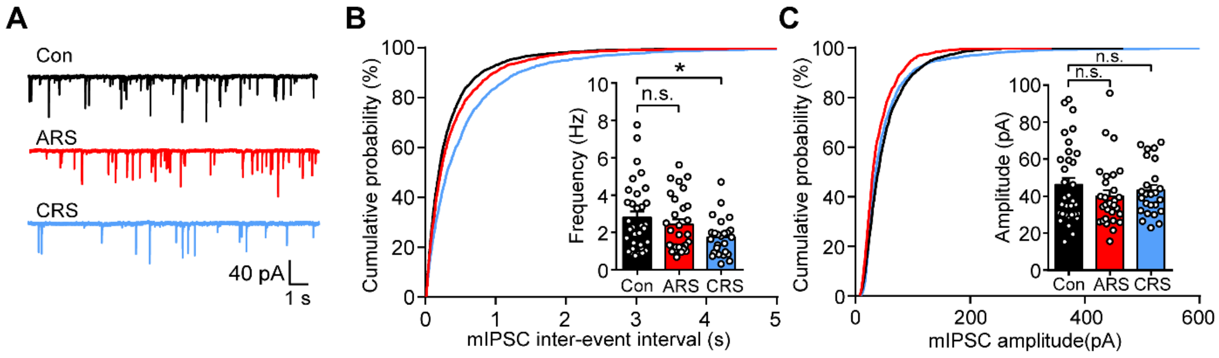

Supplementary Figure S1. CRS suppresses the GABAergic transmission of LHb neurons. (A) Representative traces of mIPSC recorded from the LHb neurons of control, ARS and CRS mice. **(B)** Summary data for mIPSC frequency recorded from the LHb neurons of control, ARS and CRS mice. **(C)** Summary data for mIPSC amplitude recorded from the LHb neurons of control, ARS and CRS mice. n = 33 neurons, from 4 mice for Con; n = 29 neurons, from 3 mice for ARS; n = 25 neurons, from 4 mice for CRS. Data are presented as the mean ± SEM. n.s., no significant difference; *$p < 0.05$, one-way ANOVA with Tukey's multiple comparisons test.

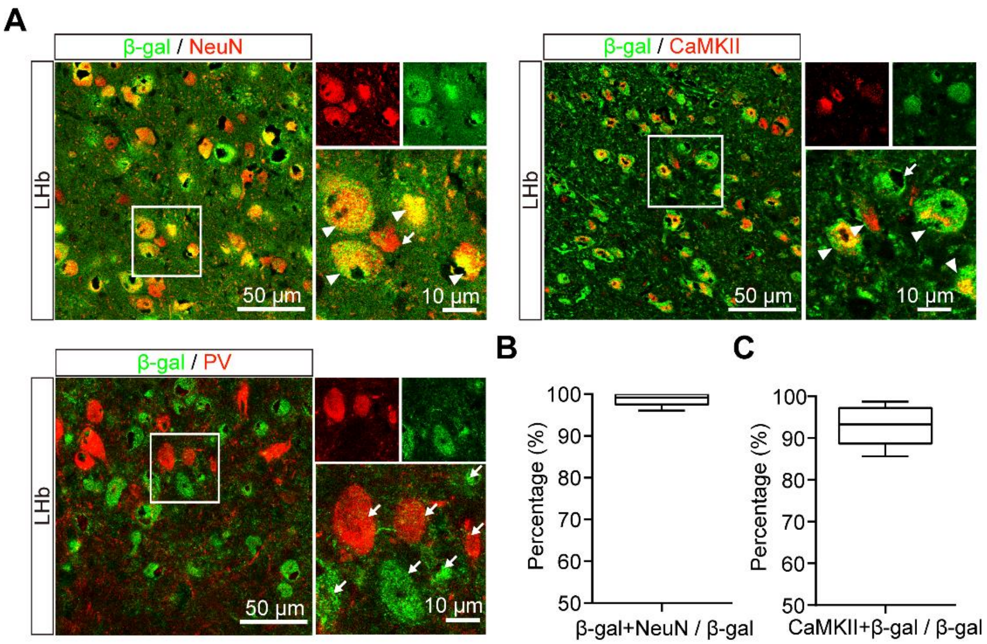

Supplementary Figure S2. MDGA1 expressed predominantly in LHb pyramidal neurons. **(A)** Double immunostaining of β-gal with neuron marker-NeuN, β-gal with pyramidal neuron marker-CaMKII, β-gal with interneuron marker-PV. **(B)** Percentage of β-gal co-localized with NeuN immunofluorescence from *Mdga1*$^{-/-}$ mice. **(E)** Percentage of β-gal co-localized with CaMKII immunofluorescence from *Mdga1*$^{-/-}$ mice. Arrowheads point to colabeled neurons whereas arrows represent singly neurons.

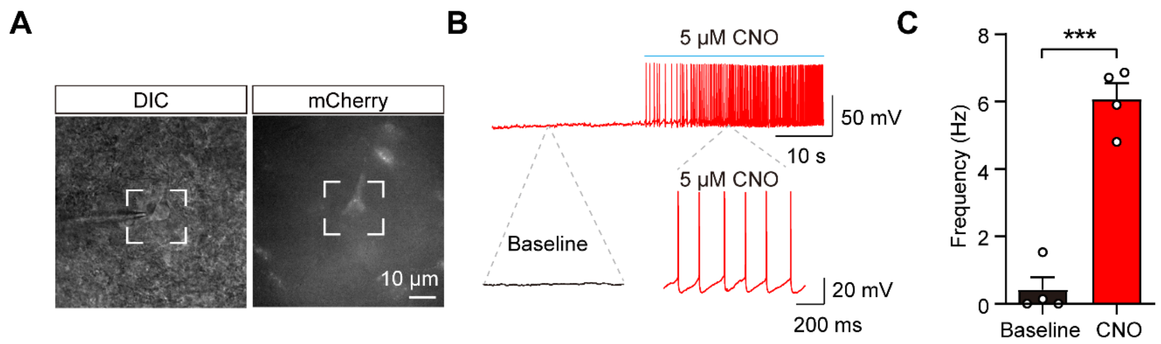

Supplementary Figure S3. Spontaneous firing rate of hM3Dq neurons in LH significantly increased upon CNO bath application. (A) Patching of viral infected LH neuron under transmitted (left) and fluorescent light microscopy (right). **(B)** Sample current-clamp traces from a LH::hM3Dq brain slice before (baseline) and during 5 mM CNO demonstrating DREADD-mediated action potentials. **(C)** CNO significantly increased spontaneous firing of LH::hM3Dq neurons (n = 4 cells from 3 mice; $p = 0.0011$); ***$p < 0.001$, unpaired t test.
